# Supplementary material for: Bacterial clustering amplifies the reshaping of eutrophic plumes around marine particles: A hybrid data-driven model
Source: PLoS Comput Biol. 2024 Dec 11;20(12):e1012660. doi: 10.1371/journal.pcbi.1012660 (PMC11666058; doi:10.1371/journal.pcbi.1012660)
Supplement: S1 Appendix — (PDF) [file pcbi.1012660.s002.pdf]

Supporting Information for

Bacterial clustering amplifies the reshaping of eutrophic plumes around marine particles: a hybrid data-driven model

George E. Kapellos*, Hermann J. Eberl, Nicolas Kalogerakis,
Patrick S. Doyle and Christakis A. Paraskeva

*Corresponding author. Email: kapellos@mit.edu

This PDF file includes:

<u>S1 Appendix.</u> RDF extraction.

Extraction of the parameters $\{\beta_0, \beta_m, n, d_s\}$ for the radial distribution function (RDF):

$$\beta(r) = \beta_0 + \beta_m \mathbf{exp}[-(r-1)^n / d_s^n] \quad \text{(S1.1)}$$

which is used to describe the bacterial concentration around nutrient-releasing particles. Here, $r = R/R_P$ is the dimensionless radial distance and R_P the particle radius. The general procedure is outlined in Figure A and applied to the following cases:

1. Bacterial RDF around a nutrient-releasing particle – synthetic data (demo, this work).
2. Bacterial RDF around an oil droplet – data by Desai & Ardekani (2018).
3. Bacterial RDF around a fecal pellet – data by Smriga et al. (2016).
4. Bacterial RDF around a Sephadex bead – data by Barbara & Mitchell (2003).
5. Bacterial RDF around an algal cell – data by Bowen et al. (1993).

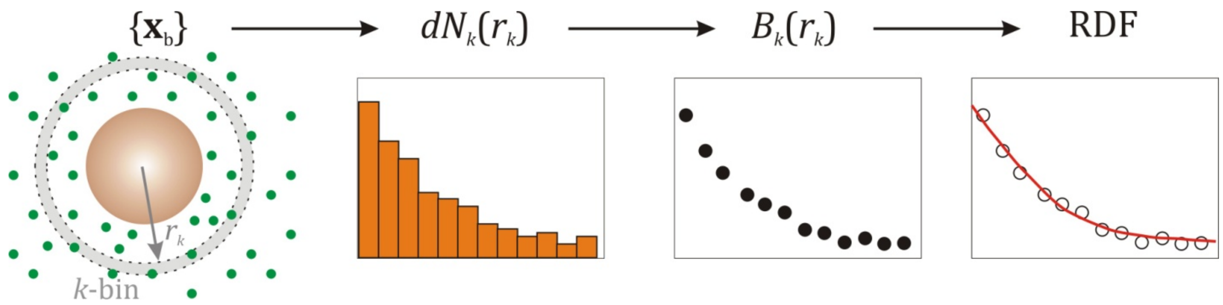

Figure A. Flowchart for RDF extraction from microscale and *in silico* experiments with single-cell resolution. <u>Step 1:</u> determination of the position vector, $\mathbf{x}_\mathrm{b}$, and radial distance $|\mathbf{x}_\mathrm{b}|$ for each cell (green dots) relative to the particle. <u>Step 2:</u> counting of cells within each interval (bin) of radial distance, dN_k. <u>Step 3:</u> calculation of discrete values for the bacterial density, B_k. <u>Step 4:</u> implementation of constrained nonlinear regression.

Section S1-1. Bacterial RDF around a nutrient-releasing particle – synthetic data (demo).

In this section, we demonstrate the general procedure for the extraction of the radial distribution function from a dataset of bacterial positions relative to a reference particle (Figure B).

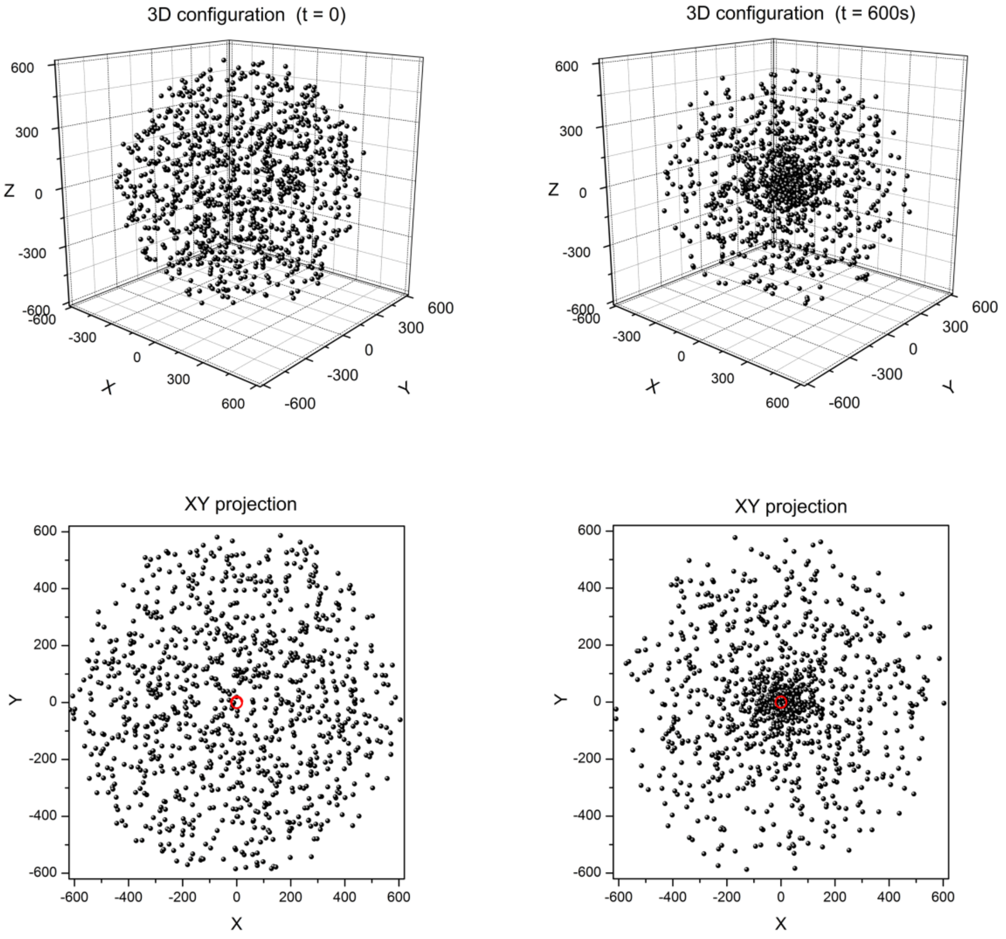

Figure B. Spatial arrangement of chemotactic bacteria around a nutrient-releasing particle, created by an individual-based simulator similar to that of *Desai et al.* (2018). Briefly, the simulation involves 1000 bacteria that perform biased random walks, of the run-and-tumble type, within a spherical domain of radius $R_\infty = 620\mu m$. A stationary particle of radius $R_P = 20\mu m$ is located at the center of the domain and releases nutrients with diffusivity $D_{Av} = 10^{-5} cm^2/s$. Initially, the bacteria are uniformly distributed (left). The configuration of clustered bacteria shown on the right is established at $600s$ and represents statistical equilibrium. The motility parameters of the bacteria are: swimming speed $V_S = 40\mu m/s$, average run time $\tau_0 = 0.6s$, ratio of nutrient concentration on the particle surface to chemoreceptor half-saturation $C_0/K_D = 0.05$, amplification time constant $a_C = 660s$, and rotational diffusivity $D_r = 0.075\ rad^2/s$.

Data acquisition (step 1). We used an individual-based simulator of bacterial chemotaxis to create the equilibrium configuration shown in Figure B for chemotactic bacteria around a stationary particle that releases chemoattractant molecules. The simulator is similar to that of *Desai et al.* (2018) and the simulation conditions are described in the figure caption. The dataset of positions, $\{\mathbf{x}_{\mathrm{b}}\}$, for 1000 bacteria was processed as follows.

Data binning (step 2). For each bacterium, the distance from the particle center was calculated, $|\mathbf{x}_{\mathrm{b}}| = \sqrt{x_b^2 + y_b^2 + z_b^2}$, and sorted into concentric spherical bins. A bacterium belongs to the *kth* bin, if the following criterion is satisfied:

$$R_{k-1} < |\mathbf{x}_{\mathrm{b}}| \leq R_k \tag{S1.2}$$

where $(R_{k-1}, R_k]$ is the radial distance interval that defines the *kth* bin. For non-spherical particles (e.g., Smriga and Barbara cases), body-conforming bins can be used for cell counting and the calculation of equivalent sphere radii.

Discrete RDFs (step 3). From the bin counts, we determine the cumulative distribution function (CDF) and the 1D, 2D, or 3D radial distribution function. The CDF is a fundamental function that is defined in continuous and discrete forms as:

$$F(R) = \frac{1}{N_\infty}\int_0^R \left(\frac{dN}{dt}\right) dt = \frac{1}{N_\infty}\int_0^R dN \tag{S1.3}$$

and

$$F_k(R_k) = \frac{1}{N_\infty}\sum_{i=1}^{k} dN_k \tag{S1.4}$$

respectively. Here, N_∞ is the total number of bacteria in the domain, and dN_k is the number of bacteria in the *kth* bin. The CDF represents the probability of finding a bacterium within the interval $(0, R]$ and relates to the RDFs as follows:

$$B_{\mathrm{1D}}(R) = N_\infty^{1D}\frac{dF}{dR} \tag{S1.5}$$

$$B_{\mathrm{2D}}(R) = \frac{N_\infty^{2D}}{2\pi R}\frac{dF}{dR} \tag{S1.6}$$

$$B_{\mathrm{3D}}(R) = \frac{N_\infty^{3D}}{4\pi R^2}\frac{dF}{dR} \tag{S1.7}$$

The RDF represents the probability of finding a bacterium at distance R from the particle center. Upon assuming that the CDF is invariant to spatial dimensions, we can estimate the 3D-RDF from 2D position data. The CDF and 3D RDF for the clustered configuration of Figure B (right) are shown in Figure C. Important features of the CDF include the ease of calculation from

discrete data (Eq. S1.4) and the insensitivity to the bin size. By fitting the CDF data to a simple function, we obtain a first continuous estimator of the RDF. For instance, we observed that a good fit to CDF data is provided by the sigmoidal Boltzmann equation:

$$F(R) = F_2 - \frac{F_2 - F_1}{1 + \mathbf{exp}((R - x_0)/dx)} \qquad \text{(S1.8)}$$

with derivative:

$$\frac{dF}{dR} = \frac{(F_2 - F_1)}{dx} \frac{\mathbf{exp}((R - x_0)/dx)}{[1 + \mathbf{exp}((R - x_0)/dx)]^2} \qquad \text{(S1.9)}$$

In the limit of $R - x_0 \gg dx$, the derivative tends asymptotically to $F_0 \mathbf{exp}[-(R - x_0)/dx]$, with $F_0 = (F_2 - F_1)/dx$, which matches the form of our excess function for the RDF with $n = 1$.

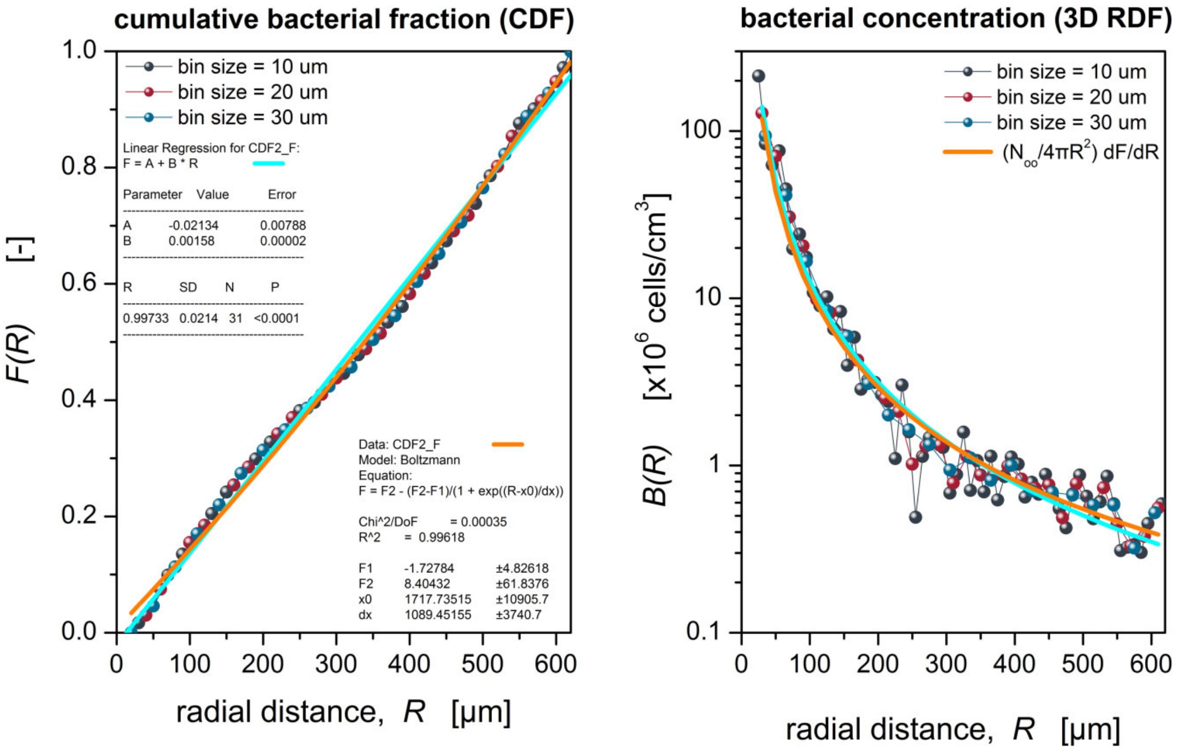

Figure C. Bacterial CDF and RDF for the configuration shown in Figure B. The cyan line corresponds to the linear function, $F(R) = A + B * R$, with $A = -0.02134$ and $B = 0.00158$, while the sigmoidal Boltzmann function, $F(R) = F_2 - (F_2 - F_1)/[1 + \mathbf{exp}((R - x_0)/dx)]$, with $F_2 = 8.4$, $F_1 = -1.7$, $x_0 = 1717$, and $dx = 1089$, is shown in orange color.

Discrete values of the RDFs can be calculated directly from bin counts (without the CDF) as follows. For the 2D RDF, the local bacterial concentration in the kth bin is calculated as $B_k = dN_k/dS_k$, where dN_k is the number of bacteria in the bin and $dS_k = \pi(R_k^2 - R_{k-1}^2)$ is the

bin area. For the 3D RDF, the local bacterial concentration in the k-th bin is calculated as $B_k = dN_k/dV_k$, where $dV_k = 4\pi(R_k^3 - R_{k-1}^3)/3$ is the bin volume.

The CDF and RDFs provide information about the internal structure of particulate systems and can be used in the statistical reconstruction of the spatial arrangement of particles for the subsequent extraction of structure-function relations.

Data regression (step 4). We used OriginLab to perform regression analysis on the dataset $\{R_k, B_k\}$ and fit the following exponential RDF:

$$B(R) = b_0 + b_1 \exp[-(R - R_0)^n / d_1^n] \tag{S1.10}$$

where R_0 is the particle size. The regression analysis is based on least-squares minimization with a Levenberg-Marquardt algorithm, subject to two constraints:

$$b_0 < B_{avg}^* \tag{S1.11}$$

$$\left|1 - B_{avg}/B_{avg}^*\right| * 100\% < 1\% \tag{S1.12}$$

Here, B_{avg} is the *estimated* average bacterial concentration that is calculated from the RDF, and B_{avg}^* is the *actual* average bacterial concentration that is known a priori from the experimental setup. The first constraint is automatically incorporated in the regression analysis by OriginLab. When necessary, the second constraint is manually enforced by fine-tuning the value of b_0 or d_1. The parameters b_0, b_1, and d_1 relate to the dimensionless parameters of Eq. (S1.1), as:

$$\beta_0 = b_0/B_{avg}^* \ ; \ \beta_m = b_1/B_{avg}^* \ ; \ d_s = d_1/R_0 \tag{S1.13}$$

Extracted parameters are given in Table A and the outcome of curve fitting in Figure D.

For this demo, we have 1000 cells in a spherical domain of radius $R_\infty = 620\mu m$, corresponding to an *actual* average concentration of $B_{avg}^* = 10^6 cells/mL$. The *estimated* average bacterial concentration is calculated from the 3D-RDF as follows:

$$B_{avg}^{RDF} = \frac{\int_{R_o}^{R_\infty} 4\pi R^2 B(R) dR}{\int_{R_o}^{R_\infty} 4\pi R^2 dR} \tag{S1.14}$$

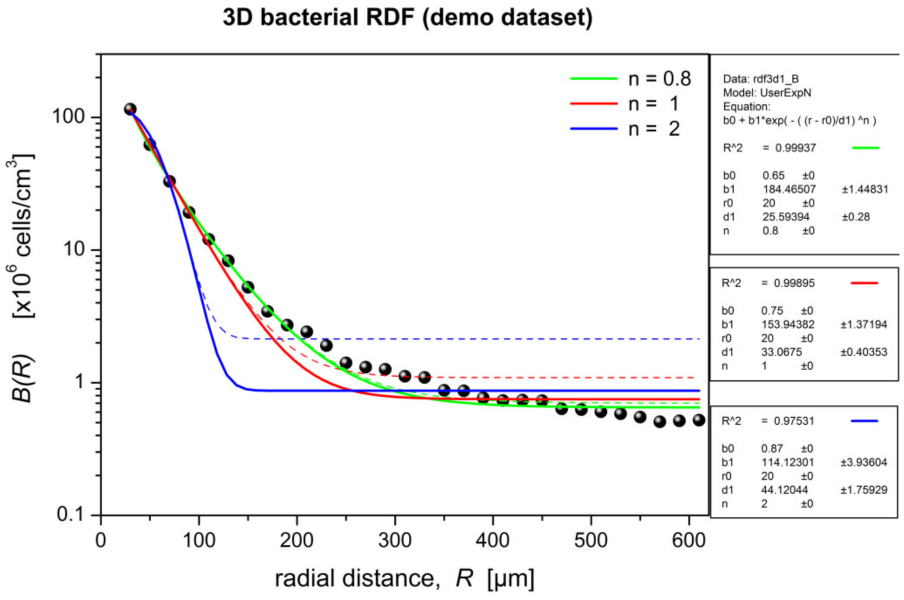

Figure D. Constrained regression analysis for the synthetic dataset. The smoothed RDF data points are time-averages of 12 configurations, sampled every 100*s* in statistical equilibrium (i.e., once the average distance of the particles from the center attains an asymptotic value). The continuous lines correspond to constrained regression and the dashed lines to unconstrained.

Table A. RDF parameters extracted with regression analysis for the demo dataset.

R_0 [μm]	n [-]	b_0 [cells/mL]	b_1 [cells/mL]	d_1 [μm]	R^2 [-]	B_{avg}^{RDF} (% R.E.) [x10^6 cells/mL]	β_0 [-]	β_m [-]	d_s [-]
**unconstrained**									
20	4/5	0.70 x10^6	184.5 x10^6	25.5	0.9994	1.04 (4%)	0.67	177	1.27
20	1	1.09 x10^6	154.1 x10^6	32.7	0.9991	1.33 (33%)	0.82	115	1.64
20	2	2.14 x10^6	113.7 x10^6	43.1	0.9778	2.3 (126%)	0.94	50	2.15
**constrained**									
20	4/5	0.65 x10^6	184.5 x10^6	25.6	0.9994	1.00 (<1%)	0.65	185	1.28
20	1	0.75 x10^6	154.0 x10^6	33.1	0.9989	1.00 (<1%)	0.75	154	1.65
20	2	0.87 x10^6	114.1 x10^6	44.1	0.9753	1.00 (<1%)	0.87	114	2.20

The fractional exponent $n = 4/5$ provides the best fit to the data, in terms of R^2 and visually, and $n = 1$ is the near-optimal integer exponent.

Observation window effects. The regression outcome and the average bacterial concentration depend on the size of the observation window, i.e., the radial distance R_∞ up to which data are provided. Extracted parameters are listed in Table B and the outcome of curve fitting is shown in Figure E, for different values of R_∞. The non-normalized peak, b_1, and the chemotaxis length, d_S, depend on the data close to the particle surface, rather than further away, and hence are independent of the observation window size. However, a small observation window results in overestimation of the average and ambient concentrations, B_{avg}^* and b_0, and underestimation of the dimensionless ambient and peak values, β_0 and β_m.

Table B. Effect of the observation window $\{R_\infty\}$ on the RDF parameters.

R_∞ [μm]	N_C [cells]	n [-]	b_0 [cells/mL]	b_1 [cells/mL]	d_1 [μm]	B_{avg}^{RDF} (%R.E.) [x10^6 cells/mL]	β_0 [-]	β_m [-]	d_s [-]
**160**	254	1	4.50 x10^6	155.1 x10^6	29.8	14.8 (<1%)	0.30	10.5	1.5
**320**	457	1	1.65 x10^6	154.4 x10^6	32.2	3.3 (<1%)	0.49	46.0	1.6
**620**	1000	1	0.75 x10^6	154.0 x10^6	33.1	1.0 (<1%)	0.75	154.0	1.6

For each observation window, the *actual* average bacterial concentration is calculated as $B_{avg}^* = N_C/(4\pi R_\infty^3/3)$, where N_C is the number of cells within the observation window.

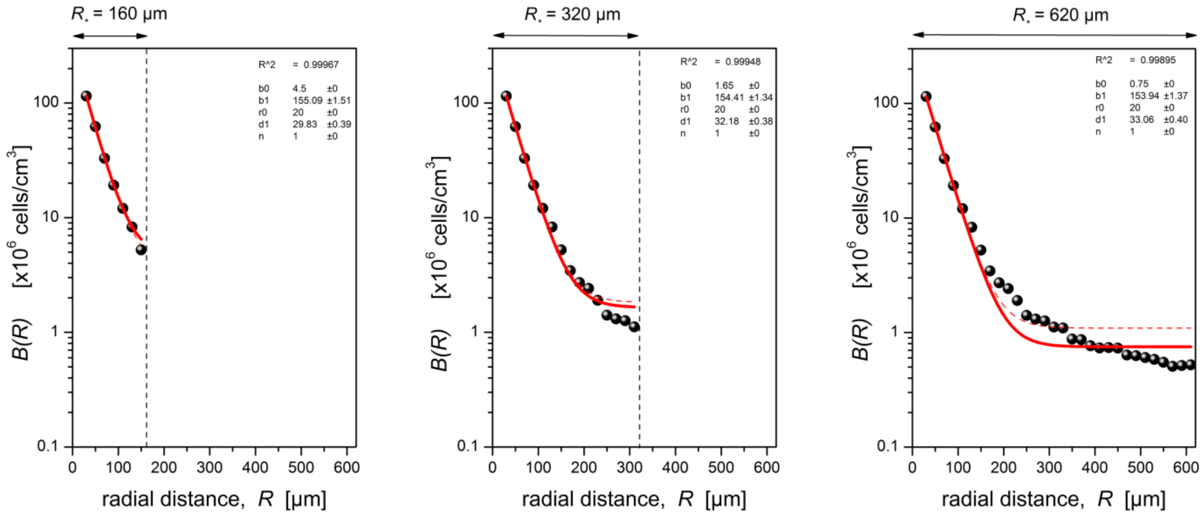

Figure E. Regression analysis of the synthetic dataset for three observation windows.

Section S1-2. Bacterial RDF around an oil droplet – data by Desai & Ardekani (2018).

Desai & Ardekani (2018) investigated computationally the accumulation of motile chemotactic bacteria around stationary nutrient-releasing particles. They studied the effects of chemotaxis and cell-particle hydrodynamic interactions on the bacterial RDF. We analyzed the results from Figure 12 in their paper for a stationary oil droplet (Figure F).

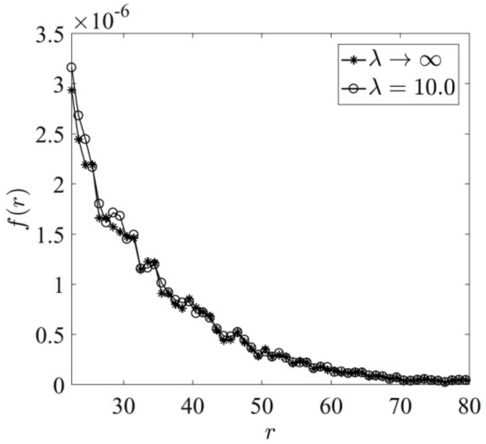

Figure F. Modified (dimensionless) radial distribution function for motile marine bacteria around a neutrally buoyant oil droplet. Reprinted from [*Phys. Rev. E* **98**, 012419, 2018], with permission from the publisher (APS) and the authors.

Data acquisition. We used WebPlotDigitizer v.4 [https://apps.automeris.io/wpd4/] to extract discrete data for the modified RDF, *f*, which relates to the bacterial density, *B*, in cells/μm^3 as $B(R) = f(R/L_b) * N_b/L_b^3$, where $L_b = 1\mu m$ is the bacterium size and $N_b = 10^4$ is the number of bacteria used in the computational experiment of Desai & Ardekani (2018). There are 58 data points $\{R_k, B_k\}$ for the discrete 3D RDF.

Data processing. We used OriginLab to perform regression analysis on the dataset $\{R_k, B_k\}$ and fit the exponential RDF defined in Equation S.10, subject to the constraints of {S.11 and S.12}. For the specific simulation, in the legend of their Fig. 12, *Desai et al.* mention that the fraction of attached bacteria was 0.3446 or, equivalently, 3446 out of a total of 10,000 bacteria. Thus, 6554 bacteria remained as free-living within the spherical observation window defined by the radii $R_0 = 20\mu m$ and $R_\infty = 80\mu m$, which corresponds to an actual average bacterial concentration of $B_{avg}^* = 3.1 \times 10^9 cells/mL$. Extracted parameters are given in Table C and the outcome of curve fitting is presented in Figure G.

Table C. RDF parameters extracted with regression analysis for the oil droplet.

R_0 [μm]	n [-]	b_0 [cells/mL]	b_1 [cells/mL]	d_1 [μm]	R^2 [-]	B_{avg}^{RDF} (% R.E.) [x10^9 cells/mL]	β_0 [-]	β_m [-]	d_s [-]
**unconstrained**									
20	4/5	0.	42.3 x10^9	9.6	0.9901	3.2 (<4%)	0.00	13.1	0.48
20	1	0.19 x10^9	34.8 x10^9	12.3	0.9877	3.1 (<2%)	0.06	11.4	0.61
20	2	1.44 x10^9	23.3 x10^9	16.1	0.9495	3.2 (<4%)	0.45	7.3	0.81
**constrained**									
20	1	0.31 x10^9	34.8 x10^9	12.1	0.9876	3.1 (<1%)	0.10	11.2	0.61
20	2	1.30 x10^9	23.4 x10^9	16.3	0.9493	3.1 (<1%)	0.42	7.5	0.82

For this dataset, the observation window is small ($R_\infty/R_P = 4$) and captures only the exponential part of the RDF. Thus, the average bacterial concentration (B_{avg}^*) is overestimated and results in underestimation of the dimensionless peak and ambient concentrations, β_m and β_0.

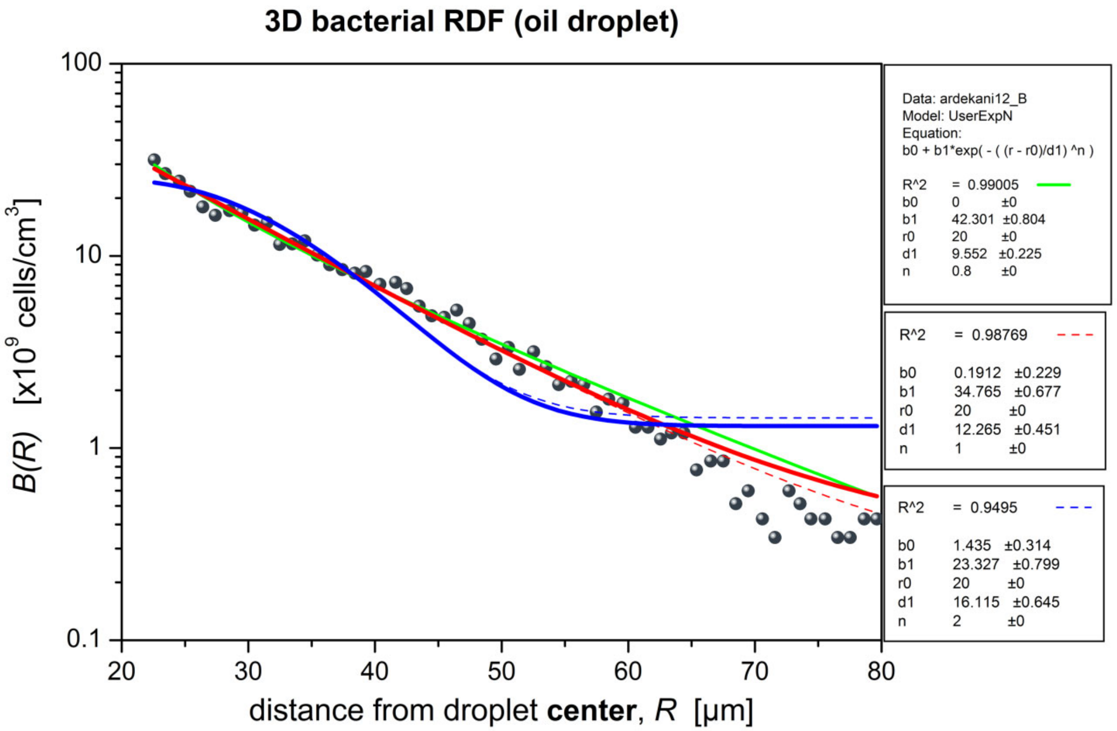

Figure G. Regression analysis of the RDF dataset for the oil droplet.

Section S1-3. Bacterial RDF around a fecal pellet – data by Smriga et al. (2016).

Smriga et al. (2016) investigated with microscale experiments the accumulation of motile marine bacteria around individual phytoplankton cells and fecal pellets. We analyzed the results from Movie S2 in their paper for a fecal pellet suspended in a drop of seawater (Figure H).

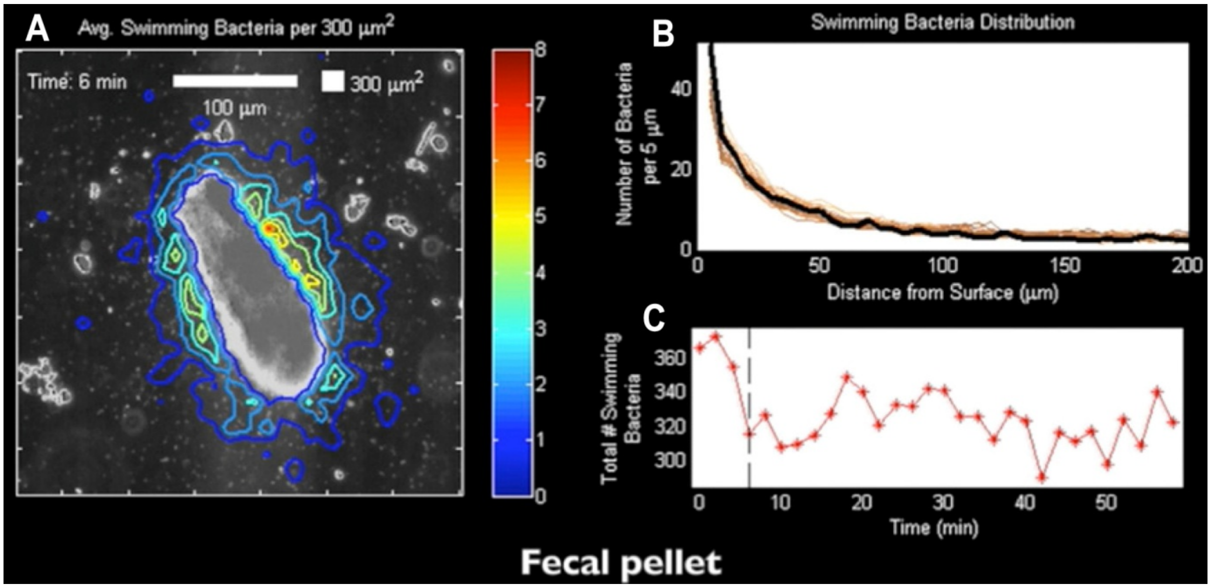

Figure H. Distribution of marine bacteria around a fecal pellet. Reprinted from Movie S2 in the SI of [*PNAS* **113**(6), 1576-1581, 2016], with permission from the publisher (PNAS) and the authors. Figure caption from source: "(**A**) Contours of cell concentration, quantified as the number of motile bacteria per area of 300 μm^2 (color bar). The bacteria are seen as white dots and the fecal pellet is delineated by a dark blue line. (**B**) Total number of motile bacteria in 5-μm-wide concentric annuli, as a function of the distance from the surface of the fecal pellet. (**C**) Total number of swimming bacteria as a function of time."

Data acquisition. We extracted the number of bacteria per bin, *dN*, from panel B of Fig H using WebPlotDigitizer v.4 [https://apps.automeris.io/wpd4/]. Bins are non-overlapping 5μm-wide concentric annuli around the fecal pellet. There are 40 data points $\{R_k, dN_k\}$ of radial binning.

Data processing. From the bin counts, we determined the cumulative distribution function (CDF) and the 1D, 2D, and 3D RDFs shown in Figure I. For the 3D RDF, we calculated the local bacterial concentration in the k-th bin as $B_k = dN_k/dV_k$, where dV_k is the bin volume calculated as $dV_k = 4\pi(R_k^3 - R_{k-1}^3)/3$ and $(R_{k-1}, R_k]$ is the radial distance interval that defines the k-th bin. The equivalent sphere radius (ESR) of the fecal pellet, $R_0 = 80\mu m$, was determined as

described below. For this dataset, we do not have information about the projected area and volume of the concentric annuli that were used in cell binning. So, the equivalent sphere radii of the bins were estimated as $ESR = R_0 + (distance\ from\ surface)$.

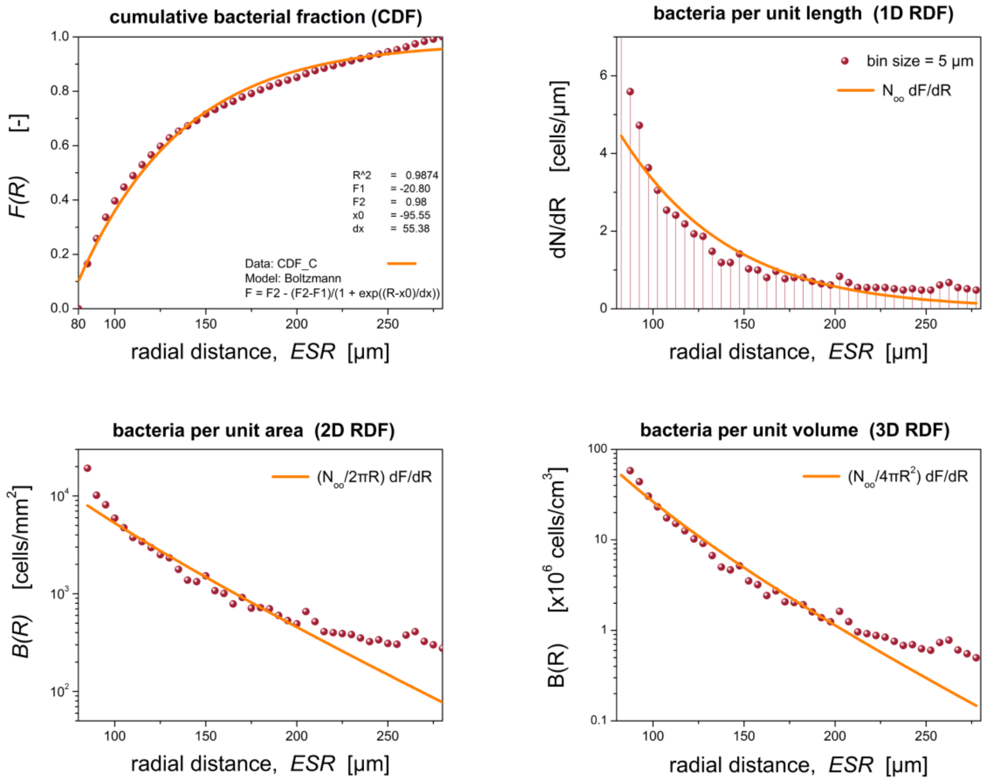

Figure I. Bacterial CDF and RDFs for the fecal pellet dataset.

Constrained regression analysis and fitting of the exponential RDF defined in Equation S.10 to the dataset $\{R_k, B_k\}$ was performed with OriginLab, as described in Section S1-1. For the actual average bacterial concentration, we considered the observation window, defined by the interval $(R_0, R_\infty]$ with $R_\infty = 280\mu m$, as a circular/spherical shell that contains 316 bacteria (panel C of Figure H) to obtain the values of 1397 cells/mm^2 in 2D and 3.5×10^6 cells/mL in 3D. The bacterial concentration in 3D is close to the experimental value reported in the reference (1.18×10^6 cells/mL; p.2 of SI by *Smriga et al.*). Extracted parameters are given in Table D and the outcome of curve fitting is presented in Figure J.

For this dataset, the optimal curve fitting is obtained for a fractional exponent of about $n = 3/5$, with a high R-squared value (>0.99), low % R.E. in the estimation of the average bacterial concentration (<5%), and very good visual fit to the data. However, in the analysis of plume quenching in the main paper, we opted to use the integer exponent $n = 1$, which provides

a reasonably good fit (R^2>0.99, <5% R.E. in B^*_{avg}, satisfactory visual fit), because we are not certain whether the fractional exponent is related to the underlying processes or to the afore-mentioned geometrical uncertainty in the estimation of the bin volume/area.

Table D. RDF parameters extracted with regression analysis for the fecal pellet.

R_0 [μm]	n [-]	b_0 [cells/mL]	b_1 [cells/mL]	d_1 [μm]	R^2 [-]	B^{RDF}_{avg} (% R.E.) [x10^6 cells/mL]	β_0 [-]	β_m [-]	d_s [-]
**unconstrained**									
80	3/5	0.52 x10^6	202.4 x10^6	6.9	0.9981	3.7 (5%)	0.14	54.5	0.09
80	1	1.30 x10^6	97.1 x10^6	17.8	0.9938	3.7 (5%)	0.35	26.3	0.22
60	1	1.80 x10^6	174 x10^6	16.1	0.9944	4.7	0.38	36.9	0.27
80	2	2.11 x10^6	57.9 x10^6	27.2	0.9645	3.9 (11%)	0.54	14.8	0.34
**constrained**									
80	3/5	0.25 x10^6	198.0 x10^6	7.15	0.9979	3.50 (<1%)	0.07	56.3	0.09
80	1	1.10 x10^6	96.5 x10^6	18.1	0.9937	3.50 (<1%)	0.31	27.4	0.23
80	2	1.70 x10^6	57.7 x10^6	27.8	0.9637	3.50 (<1%)	0.48	16.3	0.35

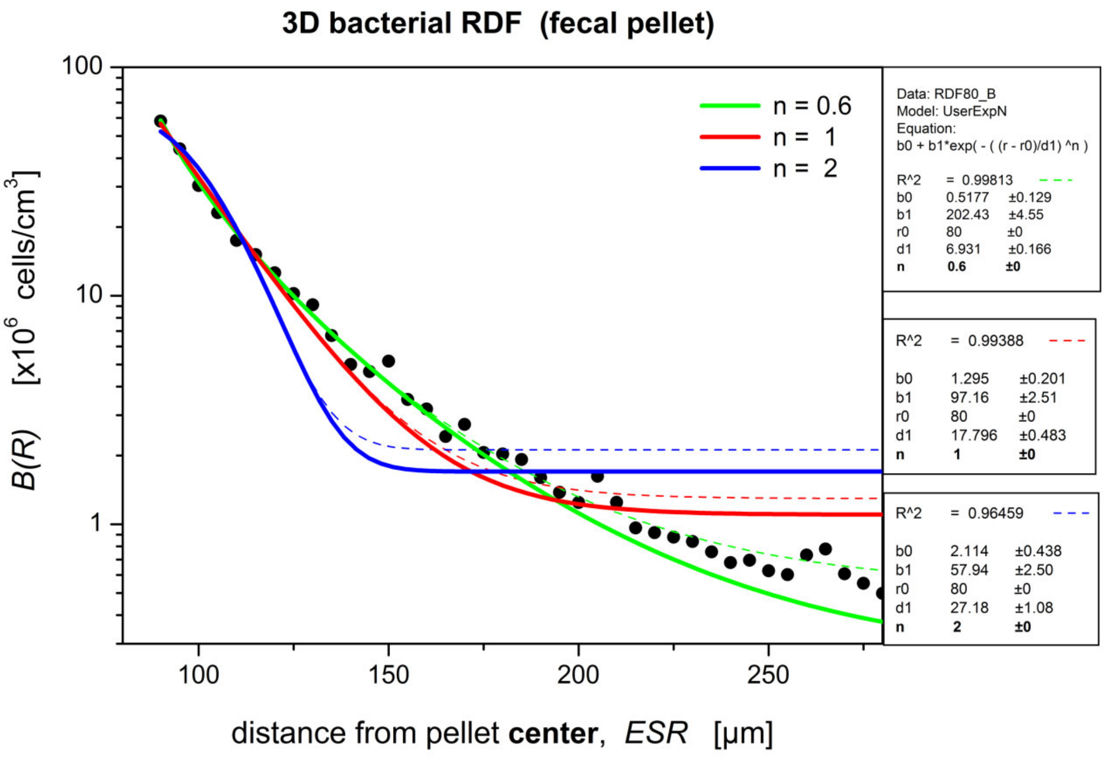

Figure J. Regression analysis of the RDF dataset for the fecal pellet.

Fecal pellet radius. First, we measured the projected area S, the projected perimeter P, and the Feret diameter D_F of the fecal pellet using image analysis (Figure K). Then, we calculated the respective equivalent radii as $R_S=sqrt(S/\pi)$, $R_P=P/(2\pi)$ and $R_F=D_F/2$. Table E presents data from three measurements. Both the projected area and Feret methods gave a radius of ~60μm, while the perimeter-based radius is ~80μm. As seen in Table D, both radii gave comparable results for the dimensionless RDF parameters. In the main text, we present the results for R_P =80μm because the dissolution process occurs through the pellet-water interface and hence the perimeter-based radius seems more suitable.

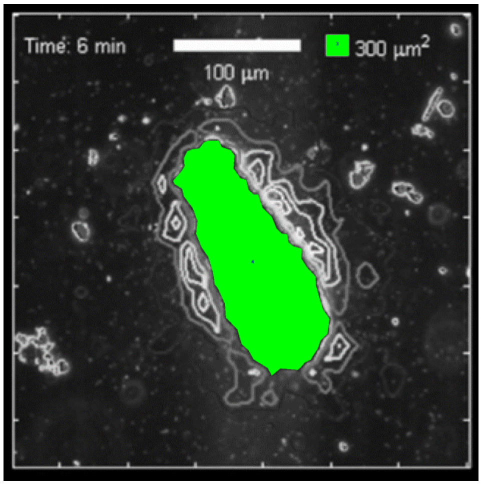

Figure K. Processed image for the determination of the projected area, perimeter and Feret diameter of the fecal pellet. The large green area represents the fecal pellet. The area of the small rectangle (300μm^2) was used to verify the spatial calibration.

Table E. Image analysis data for the equivalent disk radius of the fecal pellet.

#	S [μm^2]	P [μm]	D_F [μm]	R_S [μm]	R_P [μm]	R_F [μm]
1	11212.11	514.44	119.48	59.75	81.92	59.74
2	11209.38	517.42	119.46	59.75	82.39	59.73
3	11342.06	511.13	120.17	60.10	81.39	60.08
**average**	**11254.52**	**514.33**	**119.70**	**59.87**	**81.90**	**59.85**

Section S1-4. Bacterial RDF around a Sephadex bead – data by Barbara & Mitchell (2003). *Barbara & Mitchell* (2003) investigated experimentally the spatial organization of chemotactic marine bacteria around Sephadex beads that release amino acids. Sephadex™ is a porous gel of crosslinked dextran used in gel filtration applications (e.g., protein purification). These beads enable the controllable and reproducible binding and unloading of small molecules, like amino acids. We extracted the RDF from a micrograph (Figure 4A of the reference) that illustrates the massive accumulation of bacteria around a chemoattractant-releasing bead (Figure L).

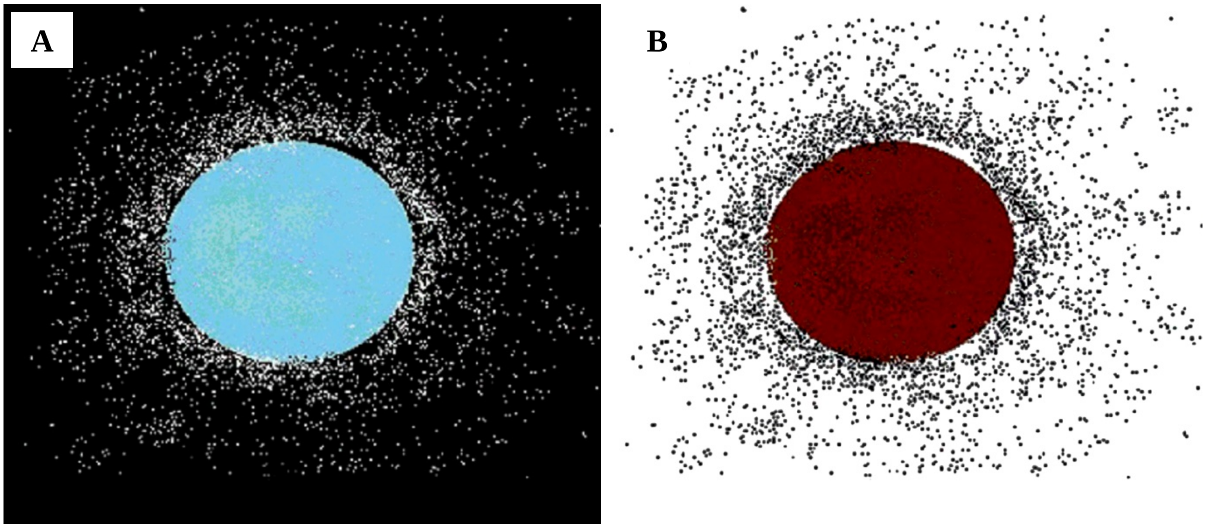

Figure L. (**A**) Accumulation of chemotactic bacteria (white dots) around a Sephadex bead that releases amino acids (leucine). Reprinted from [*FEMS Microbiol. Ecol.* **43**, 99-109, 2003], with permission from the publisher (Oxford University Press) and the authors. (**B**) Processed image with increased contrast between background and bacteria (black dots) that was analyzed to determine the position of each bacterium relative to the particle center.

Pre-processing (contrast enhancement). The original image has low contrast between cells and background. To facilitate image processing, we enhanced the contrast in Photoshop. The original image was inverted, the resolution set to 600dpi, the contrast increased (+24), and the highlights darkened by 50% (this last step increased the contrast between the center and the periphery of each cell). The center of the bead was determined and marked with yellow calibration lines.

Data acquisition. The position (x_b, y_b) of each bacterium relative to the center of the bead was determined using WebPlotDigitizer (Figure M). In spite of the enhanced contrast, many bacteria were touching each other and was difficult to tell apart, especially for those in close proximity to

the particle surface. This was in part tackled by setting the magnification to closely match the pointer size with the bacterium size in the zoom view of the software (right panel in Figure M). We measured manually the position of 2105 bacteria.

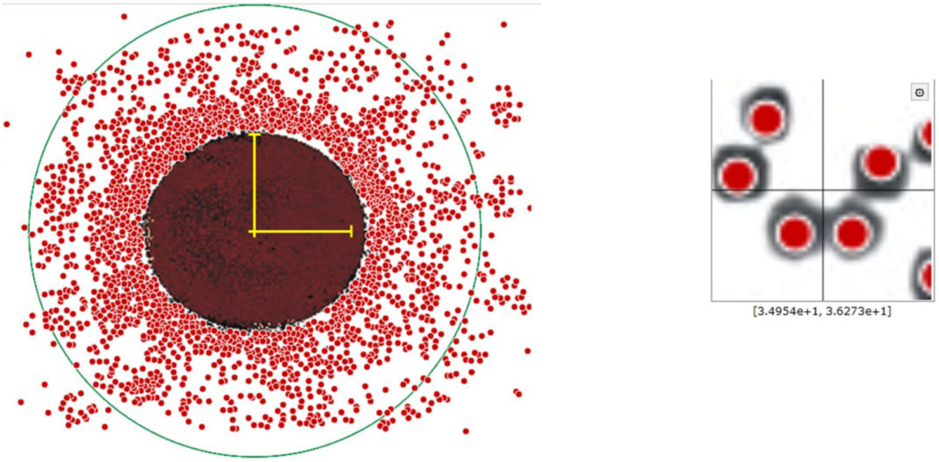

Figure M. Extraction of position data from Figure L with WebPlotDigitizer. The yellow calibration lines are equal to the length of the minor y-semiaxis of the bead and cross at the bead center. The red dots are calibrated data points sampled at the centers of bacteria. The green circle has a characteristic radius of $R_g = 2.34 * R_0$, such that all bacteria are contained between two parallel lines of $y = \pm R_g$. The green circle contains >90% of the bacteria and is used to calculate a maximum estimate for the average bacterial concentration around the particle.

Elliptical binning. The particle is slightly elliptical with minor y-semiaxis length $b_0 = 30\mu m$, major x-semiaxis length $a_0 = 34\mu m$, and an equivalent sphere radius (ESR) of $R_0 = 32\mu m$ (same projected area). The domain around the particle was discretized with concentric elliptical bins of constant aspect ratio (15:17). For each bacterium, the distance from the bead center was calculated, $d_b = \sqrt{x_b^2 + y_b^2}$, and sorted into the k-th elliptical bin, based on the criterion:

$$\rho_{k-1}(\theta_b) < d_b \leq \rho_k(\theta_b) \quad \text{(S1.15)}$$

with
$$\rho_k(\theta_b) = a_k b_k / \sqrt{(b_k \cos\theta_b)^2 + (a_k \sin\theta_b)^2} \quad \text{(S1.16)}$$

where $\cos\theta_b = x_b/d_b$, $\sin\theta_b = y_b/d_b$, a_k and b_k are the major and minor semiaxis lengths of the k-th ellipse, respectively, and $\rho_k(\theta_b)$ is the radius of the k-th ellipse at θ_b angle.

Discrete RDF. From the bin counts, we determined the cumulative distribution function (CDF) and the 1D, 2D, and 3D RDFs shown in Figure N. For the 3D RDF, we calculated the local bacterial concentration in the k-th bin as $B_k = dN_k/dV_k$, where dN_k is the number of bacteria in the bin, dV_k is the bin volume calculated as $dV_k = 4\pi(R_k^3 - R_{k-1}^3)/3$, and $(R_{k-1}, R_k]$ is the ESR interval that defines the bin with $R_k = \sqrt{a_k b_k}$.

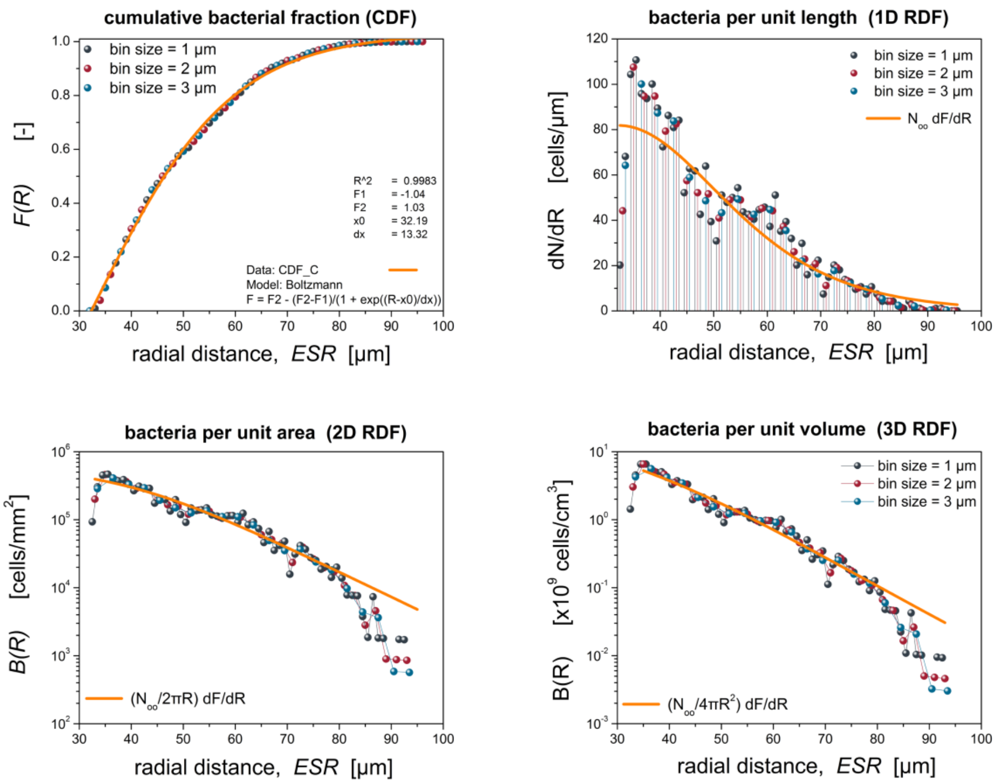

Figure N. Bacterial CDF and RDFs for the amino-acid bead dataset.

Constrained regression analysis and fitting of the exponential RDF defined in Equation S.10 to the dataset $\{R_k, B_k\}$ was performed with OriginLab, as described in Section S1-1. For the actual average bacterial concentration, we considered the observation window, defined by the interval $(R_0, R_\infty]$ with $R_0 = 32\mu m$ and $R_\infty = 90\mu m$, as a circular/spherical shell that contains 2102 bacteria to obtain the values of 94,557 cells/mm^2 in 2D and 0.72×10^9 cells/mL in 3D. The 3D value is on the same order of magnitude with experimental values (Table 1 in *Barbara et al.*). Extracted parameters are given in Table F and the outcome of curve fitting is presented in Fig O. For the regression, we excluded data points in the underflow bin (<34μm) due to interference with the particle surface and the overflow bin (>94μm) due to dilution.

Table F. RDF parameters extracted with regression analysis for the Sephadex bead.

R_0 [μm]	n [-]	b_0 [cells/mL]	b_1 [cells/mL]	d_1 [μm]	R^2 [-]	B_{avg}^{RDF} (% R.E.) [x10^9 cells/mL]	β_0 [-]	β_m [-]	d_s [-]
**unconstrained**									
32	4/5	0.	1.4 x10^9	8.1	0.9903	0.80 (12%)	0	13.0	0.25
32	1	0.05 x10^9	8.5 x10^9	10.5	0.9896	0.77 (7%)	0.07	11.1	0.32
32	2	0.31 x10^9	5.8 x10^9	13.4	0.9608	0.79 (10%)	0.39	7.4	0.42
**constrained**									
32	1	0.05 x10^9	9.1 x10^9	9.6	0.9870	0.72 (<1%)	0.07	12.5	0.30
32	2	0.22 x10^9	5.8 x10^9	13.9	0.9593	0.72 (<1%)	0.30	8.1	0.43

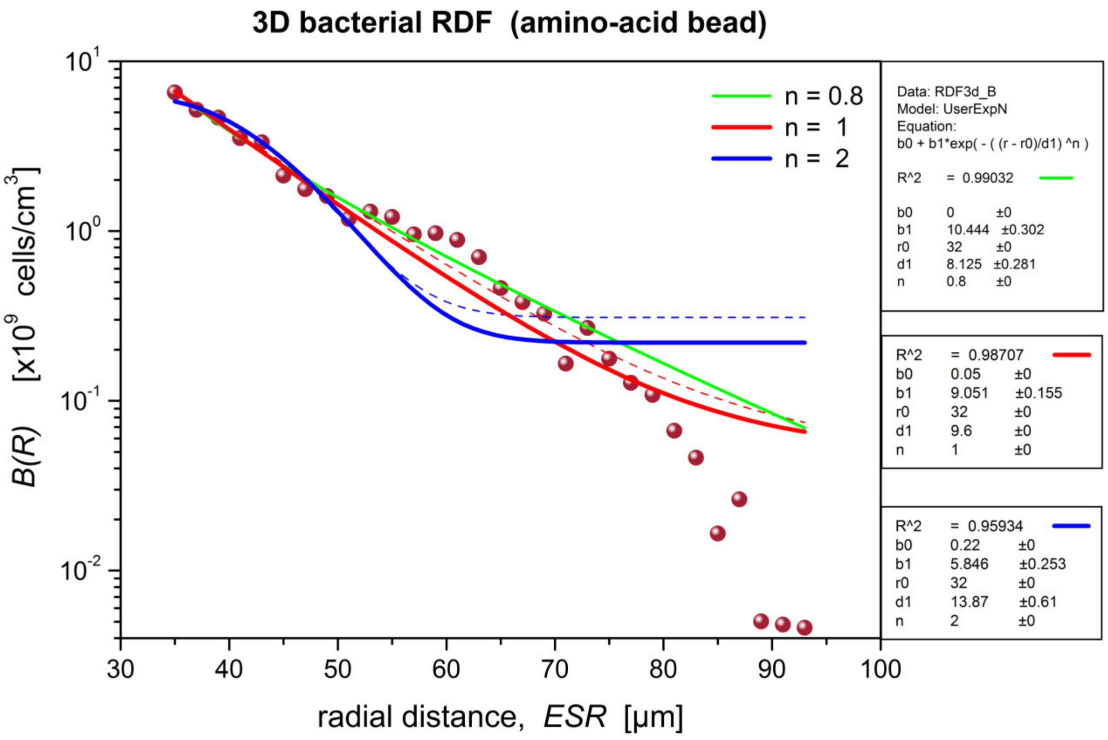

Figure O. Regression analysis of the RDF dataset for the Sephadex bead.

Uncertainty in bacterial count. We probably underestimate the bacterial concentration close to the particle due to cell clustering and cell-cell overlap. The number of overlapping bacteria along the focal depth (direction normal to image plane) cannot be assessed in the projected 2D image. However, the error associated with manual counting of bacteria, especially in clusters, can be

partially assessed by estimating the bacterial number with automated image analysis. First, the bead was painted white and the image was converted to grayscale in Photoshop (Figure P). Next, the number of objects (N_t) and their total surface area (S_t) was measured with image analysis software (Mocha, Jandel Scientific). An object is defined as an isolated darker-than-background area in the image. The number of objects (N_t) serves only as a lower bound for the number of bacteria because clusters may be identified as single objects. Another estimate for the number of bacteria in the image was obtained by dividing the total surface area of all objects (S_t) by the average surface area of isolated bacteria (S_b). These measurements require to define a threshold for the intensity of gray in the range from black (0) to white (255). Pixels with gray intensity less than the threshold belong to objects, and otherwise to background.

As seen in Figures P and Q, the threshold value has a strong effect on the results. At low threshold values (<40), objects with high intensity (i.e., closer to white) are missed and the surface area of single-cells is underestimated. At high threshold values (>64), objects in close proximity cannot be distinguished and the total surface area is overestimated. A near optimal range of threshold values (40-64) minimizes the difference between N_t and S_t/S_b and provides good resolution of touching objects and surface area. In this range, the deviation from manually counted bacteria was less than 6% (Table G).

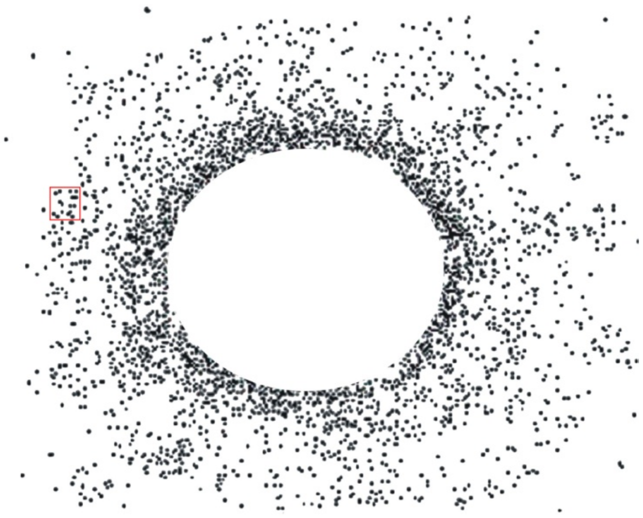

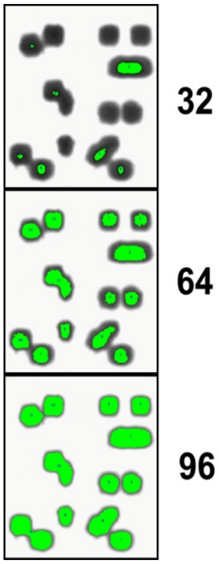

Figure P. (*Left*) Adjusted image for automatic measurement of the number and surface area of objects (bacteria and clusters). (*Right*) Effect of threshold on object identification.

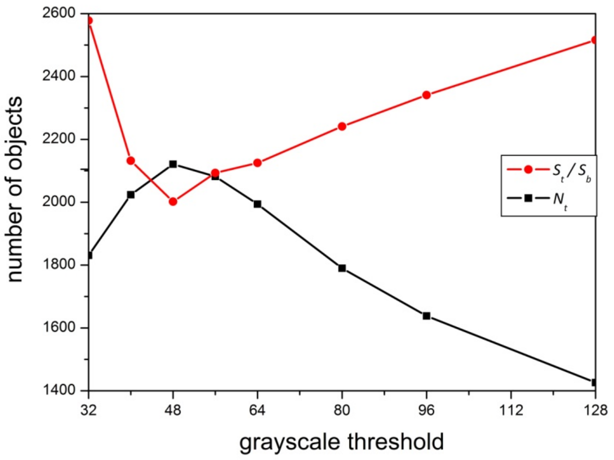

Figure Q. Effect of the grayscale threshold on the total number of objects (bacteria and clusters) around the bead.

Table G. Total number of objects (N_t), total surface area (S_t) and single-object surface area (S_b) in Figure P. Deviation from the manual count of 2105 bacteria is given in parentheses.

Threshold	N_t (%dev)	S_t [px^2]	S_b [px^2]	S_t/S_b (%dev)
32	1831 (13.%)	47697	18.52	2575 (22.%)
40	2024 (3.8%)	57902	27.15	2132 (1.3%)
48	2121 (0.8%)	81688	40.79	2002 (4.9%)
56	2082 (1.1%)	119575	57.11	2093 (0.6%)
64	1994 (5.3%)	144333	67.89	2125 (0.9%)
80	1790 (15.%)	191323	85.36	2241 (6.4%)
96	1638 (22.%)	231467	98.84	2341 (11.%)

Sephadex bead radius. There is some uncertainty about the bead size because *Barbara et al.* provided this figure only for qualitative comparison and without a scale bar. The size (diameter) of Sephadex G-50 superfine beads is in the range of 20−50μm when the particles are dry and 20−80μm when the particles are wet (product specs, Cytiva Life Sciences). In their calculations, *Barbara & Mitchell* considered a bead radius of 15μm (p.108), which is on the low end of wet bead sizes. However, from Figure 5 in their paper, which has a scale bar and shows part of a bead, we estimated a bead radius of about 36μm ± 3μm by assuming circular geometry and relating the measured arc and chord lengths for the shown part of the bead.

Section S1-5. Bacterial RDF around an algal cell – data by Bowen et al. (1993).

Bowen et al. (1993) investigated computationally the spatial organization of chemotactic marine bacteria around nutrient-exuding algal cells under shear flow that is generated at the cell scale by macroscopic turbulence. They studied the effects of fluid shear strength, nutrient exudation rate (F^*), and bacterial swimming velocity (V_S) on the bacterial distribution. We analyzed the results from Figures 5, 2A, and 2C in their paper (Figure R).

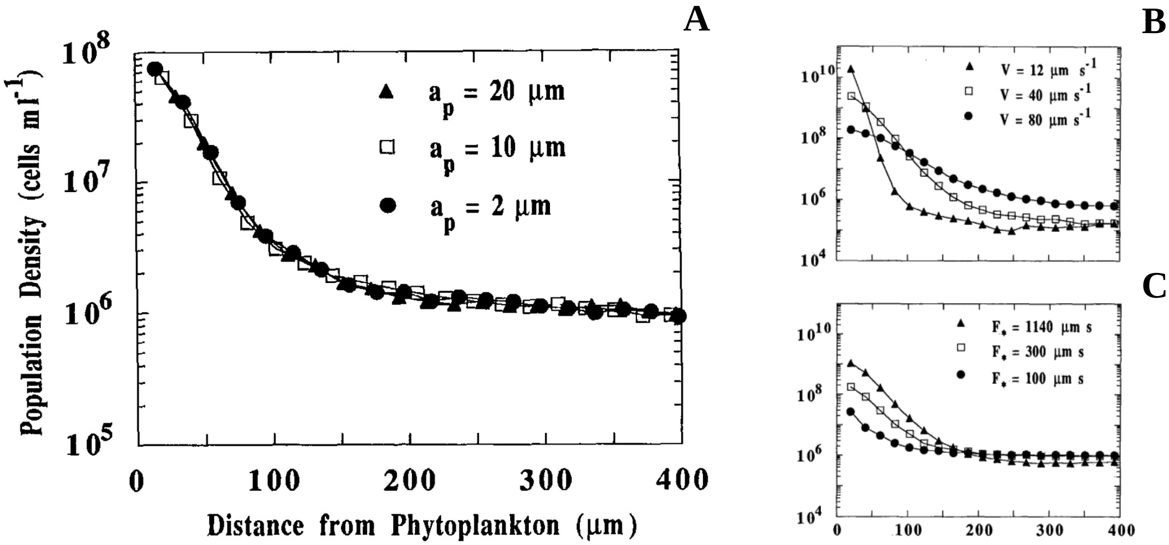

Figure R. Radial distribution functions for chemotactic marine bacteria around an algal cell. Reprinted from *Bowen et al.* [*Limnol. Oceanogr.* **38**(1), 36-51, 1993], with permission from the publisher (Wiley) and the authors.

Data acquisition. We used WebPlotDigitizer v.4 [https://apps.automeris.io/wpd4/] to extract discrete data for the bacterial concentration, *B*, in cells/cm^3, from the original graphs.

Data processing. Constrained regression analysis and fitting of the exponential RDF defined in Equation S.10 to the datasets $\{R_k, B_k\}$ was performed with OriginLab, as described in Section 1. In the simulations of *Bowen et al.*, the observation window is a sphere of radius $R_\infty = 620\mu m$ that contains 1 algal cell fixed at the sphere center, and 1000 bacteria performing biased random walks (run-and-tumble). The actual average bacterial concentration is $B^*_{avg} = 10^6 cells/mL$. Extracted parameters are given in Tables H, I, J and the outcome of curve fitting is presented in Figures S, T, and U.

Table H. RDF parameters extracted with regression analysis from panel A in Figure R for an algal cell of radius $R_0 = 20\mu m$, bacteria with swimming speed $V_S = 40\mu m/s$, and a normalized nutrient exudation rate of $F^* = 200\mu m \cdot s$.

R_0 [μm]	n [-]	b_0 [cells/mL]	b_1 [cells/mL]	d_1 [μm]	R^2 [-]	B_{avg}^{RDF} (% R.E.) [x10^6 cells/mL]	β_0 [-]	β_m [-]	d_s [-]
**unconstrained**									
20	1	1.17 x10^6	68.0 x10^6	22.7	0.9994	1.21 (21%)	0.96	56.0	1.14
20	2	1.56 x10^6	47.2 x10^6	32.3	0.9907	1.59 (60%)	0.98	29.7	1.61
**constrained**									
20	1	0.95 x10^6	67.8 x10^6	23.1	0.9991	1.00 (<1%)	0.95	68.0	1.15
20	2	0.97 x10^6	47.5 x10^6	33.0	0.9880	1.00 (<1%)	0.97	47.5	1.65

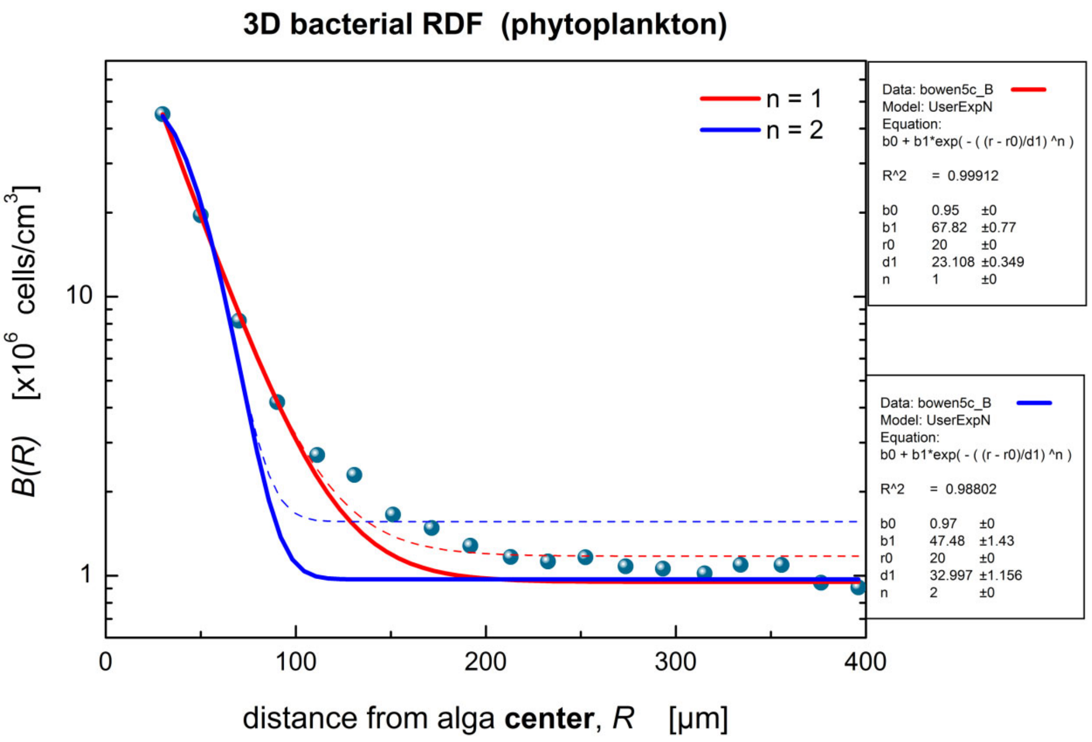

Figure S. Regression analysis of the RDF dataset from panel A in Fig R for $R_0 = 20\mu m$.

Table I. RDF parameters extracted with constrained regression analysis from panel B in Fig R for an algal cell of radius $R_0 = 10\mu m$ and three values of bacterial swimming speed (V_S).

V_S [μm/s]	n [-]	b_0 [cells/mL]	b_1 [cells/mL]	d_1 [μm]	R^2 [-]	B_{avg}^{RDF} (% R.E.) [x10^6 cells/mL]	β_0 [-]	β_m [-]	d_s [-]
12	3/2	0.05 x10^6	15,947 x10^6	12.8	~1	0.95 (~5%)	0.05	16,662	1.28
40	3/2	0.12 x10^6	1,971 x10^6	31.2	~1	0.98 (~2%)	0.12	2,011	3.12
80	3/2	0.50 x10^6	199 x10^6	62.5	~1	1.03 (~4%)	0.48	191	6.25

Table J. RDF parameters extracted with constrained regression analysis from panel C in Fig R for an algal cell of radius $R_0 = 10\mu m$ and three values of normalized exudation rate (F^*).

F^* [μm·s]	n [-]	b_0 [cells/mL]	b_1 [cells/mL]	d_1 [μm]	R^2 [-]	B_{avg}^{RDF} (% R.E.) [x10^6 cells/mL]	β_0 [-]	β_m [-]	d_s [-]
100	1/2	1.00 x10^6	128 x10^6	3.8	~1	1.03 (~3%)	0.97	125	0.38
300	5/4	0.95 x10^6	228 x10^6	30.4	~1	1.08 (~8%)	0.88	212	3.04
1140	3/2	0.50 x10^6	1013 x10^6	33.1	~1	1.01 (~1%)	0.50	1000	3.31

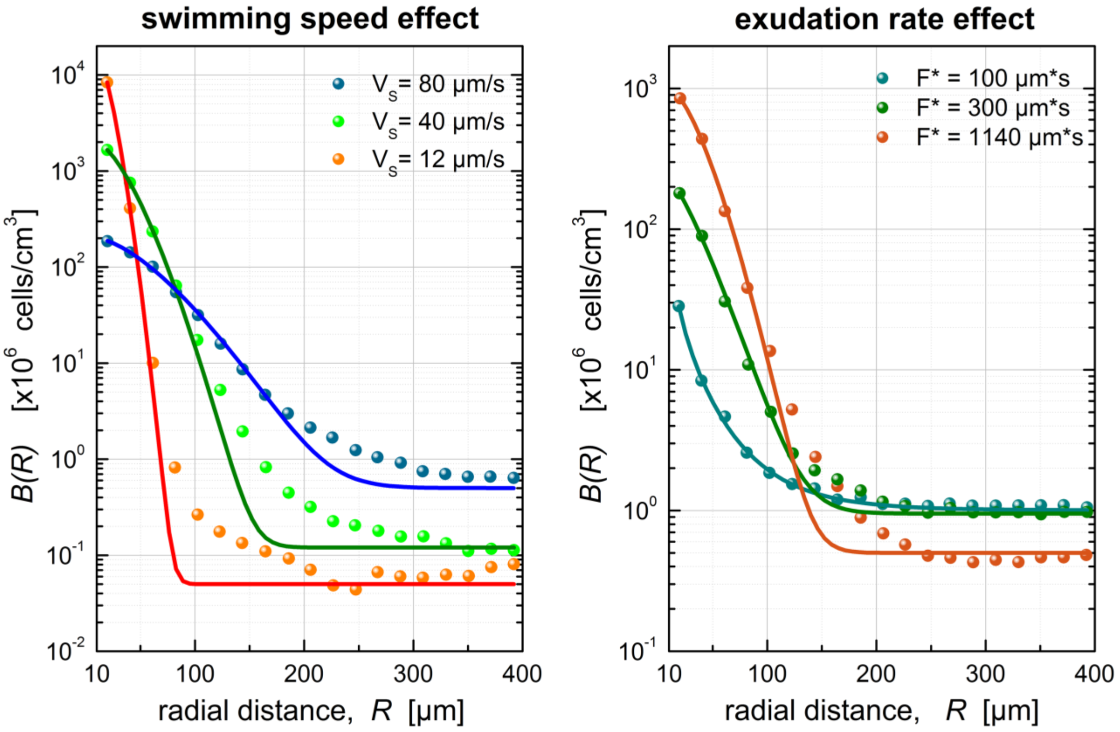

Figure T. Regression analysis of RDF datasets from panels B and C in Fig R.

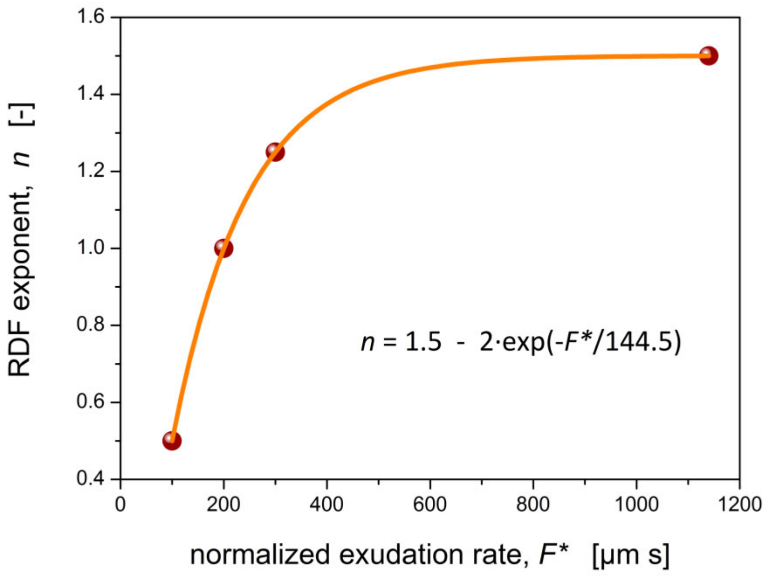

Figure U. Correlation of the RDF exponent (n) to the normalized exudation rate, for algal cells exposed to low shear strength (from 0 to 0.1 s^{-1}) and bacteria with a swimming speed of $V_S = 40\mu m/s$. The normalized exudation rate, F^* $[\mu m \cdot s]$ in Bowen's notation, captures the combined effects of the algal nutrient release and the bacterial chemotactic strength. The nutrient release rate affects the thickness of the concentration boundary layer around the algal cell, while the chemotaxis parameters weigh in the ability of the bacteria to maneuver and focus within the nutrient-rich layer. For a given flow regime, thicker boundary layers support thicker microzones. In this graph, three data points are from Table J and another ($F^* = 200\mu m \cdot s$, $n = 1$) from Table H. The continuous orange line is an exponential fit performed in OriginLab.

References

[1] G. M. Barbara, J. G. Mitchell, Marine bacterial organisation around point-like sources of amino acids, *FEMS Microbiol. Ecol.* 43(1), 99-109 (2003).

[2] J.D. Bowen, K.D. Stolzenbach, S.W. Chisholm, Simulating bacterial clustering around phytoplankton cells in a turbulent ocean, *Limnol. Oceanogr.* 38(l), 36-51 (1993).

[3] Cytiva Life Sciences, Sephadex™ product specifications. (link accessed on August 2021) https://www.cytivalifesciences.com/en/us/shop/chromatography/resins/size-exclusion/sephadex-g-50-superfine-p-05487

[4] N. Desai, A.M. Ardekani, Combined influence of hydrodynamics and chemotaxis in the distribution of microorganisms around spherical nutrient sources, *Phys. Rev. E* 98, 012419 (2018).

[5] A. Rohatgi, WebPlotDigitizer (version 4) [Computer software] (2024). Retrieved from: https://apps.automeris.io/wpd4/

[6] S. Smriga, V.I. Fernandez, J.G. Mitchell, R. Stocker, Chemotaxis toward phytoplankton drives organic matter partitioning among marine bacteria, *Proc. Natl. Acad. Sci. U.S.A.* 113, 1576-81 (2016).

```
!*************************************************************************************************
!*    *** EXTRACTION OF THE RADIAL DISTRIBUTION FUNCTION (RDF) FROM POSITION DATA ***     *
!*-----------------------------------------------------------------------------------------------------------------------------*
         PROGRAM RDF_EXTRACTION_MITCHELL
         IMPLICIT NONE
         REAL*8, PARAMETER:: scale = (45.00d0/30.0d0)   ![-], (set radius)/(reference radius)
         REAL*8, PARAMETER:: Rp = scale*30.00d0         ![um], source radius
         REAL*8, PARAMETER:: R0 = scale*30.00d0         ![um], bin start radius
         REAL*8, PARAMETER:: R1 = scale*102.0d0         ![um], bin endin radius
         REAL*8, PARAMETER:: Rg = scale*70.00d0         ![um], green circle radius  ---> average conc
         REAL*8, PARAMETER:: bin_size = scale*2.0d0     ![um], thickness of spherical shell ('bin')

!*local params-------------------------------------------------------------------------------*
         INTEGER,ALLOCATABLE:: bin_kount(:)
         REAL*8, ALLOCATABLE:: dis_data(:), Rbin(:)
         INTEGER:: ip, Np, ibin, Nb, kount1, kount2
         REAL*8 :: pi, xp, yp, dis, rm, dV, dr, dN, Bmax, Bavg1, Bavg2

         pi = 4.0d0*datan(1.0d0)

!*Determine the number of data points ---------------------------------------------------*
         OPEN(11,file="mitchell.dat",action="read")
         Np = 0
         loop: DO
           READ(11,*,END=10) xp, yp
           Np = Np + 1
         END DO loop
10       CONTINUE
         print*, "number of data ", Np
         REWIND(11)

!*Load the position data -----------------------------------------------------------------*
         ALLOCATE(dis_data(Np))
         DO ip=1,Np
           READ(11,*) xp, yp
           dis_data(ip) = scale*dsqrt( xp**2 + yp**2 )
         END DO
         CLOSE(11)

         print*, "distance min [um] ", minval(dis_data)
         print*, "distance max [um] ", maxval(dis_data)
         print*, ""

!*Create the bins -------------------------------------------------------------------------*
         Nb = NINT( (R1 - R0)/bin_size )
         ALLOCATE(Rbin(0:Nb), bin_kount(Nb))

         Rbin(0) = R0
         DO ibin=1,Nb
           Rbin(ibin) = R0 + dfloat(ibin)*bin_size
         END DO
         print*, "number of bins ", Nb
         print*, "bin size ", bin_size
         print*, ""

!OPEN(13,file='trial.dat',action='read')
!        DO i=2,22
!          READ(13,*) R_bin(i)
!        END DO

!*"Count the beans in the bins" ----------------------------------------------------------*
         bin_kount = 0
         iloop:DO ip=1,Np
           DO ibin=1,Nb
                  IF(dis_data(ip)>=Rbin(ibin-1).AND.dis_data(ip)<Rbin(ibin)) THEN
                    bin_kount(ibin) = bin_kount(ibin) + 1
                    CYCLE iloop
                  END IF
           END DO
         END DO iloop

!*Extract the RDF/CDF --------------------------------------------------------------------*
         OPEN(12,file="RDF45.dat",status='REPLACE')
         OPEN(13,file="CDF.dat",status='REPLACE')
         WRITE(13,*) Rbin(0), 0, 0.0d0
         Bmax = 0.0d0
         DO ibin=1,Nb
           dr = Rbin(ibin) - Rbin(ibin-1)                                   !----- bin thickness
           dV = 4.0d0*pi*( Rbin(ibin)**2 )*dr
           dV = 4.0d0*pi*( Rbin(ibin)**3 - Rbin(ibin-1)**3 )/3.0d0          !----- bin volume

           rm = ( Rbin(ibin) + Rbin(ibin-1) )/2.0d0                         !----- mid radius
           dN = dfloat( bin_kount(ibin) )                                   !----- bin count
           !WRITE(12,*) rm, dN/dV
           WRITE(12,*) Rbin(ibin), dN/dV
           IF(dN/dV>Bmax) Bmax = dN/dV

           kount1 = sum(bin_kount(1:ibin))
           WRITE(13,*) Rbin(ibin), kount1, dfloat(kount1)/dfloat(Np)
         END DO
         CLOSE(12)  ;  CLOSE(13)
         print*, "suspended fraction, Cf [-]  ", kount1, dfloat(kount1)/dfloat(Np)
         print*, "maximum density [cells/um3] ", Bmax


!*Average bacterial concentration in the microzone ('green circle') ---------------------------------*
         kount2 = 0
         DO ip=1,Np
           IF(dis_data(ip)>=Rp.AND.dis_data(ip)<Rg) kount2 = kount2 + 1
         END DO
         dV = 4.0d0*pi*( Rg**3 - Rp**3 )/3.0d0                               !----- total volume
         Bavg1 = dfloat(kount2)/dV

         print*, ""
         print*, "average density 1 [cells/um3] ", Bavg1
         print*, "average density 1 [cells/cm3] ", Bavg1*1.0d12
         print*, "max density ratio 1 ", Bmax/Bavg1


         dV = 4.0d0*pi*( Rbin(Nb)**3 - R0**3 )/3.0d0                         !----- total volume
         Bavg2 = sum(bin_kount(1:Nb))/dV                                     !----- underestimate
         print*, ""
         print*, "average density 2 [cells/um3] ", Bavg2
         print*, "max density ratio 2 ", Bmax/Bavg2
         print*, ""


         STOP
         END PROGRAM RDF_EXTRACTION_MITCHELL
```
