# Supplementary material for: Bacterial clustering amplifies the reshaping of eutrophic plumes around marine particles: A hybrid data-driven model
Source: PLoS Comput Biol. 2024 Dec 11;20(12):e1012660. doi: 10.1371/journal.pcbi.1012660 (PMC11666058; doi:10.1371/journal.pcbi.1012660)
Supplement: S2 Appendix — (PDF) [file pcbi.1012660.s003.pdf]

S2 Appendix: Supporting figures.

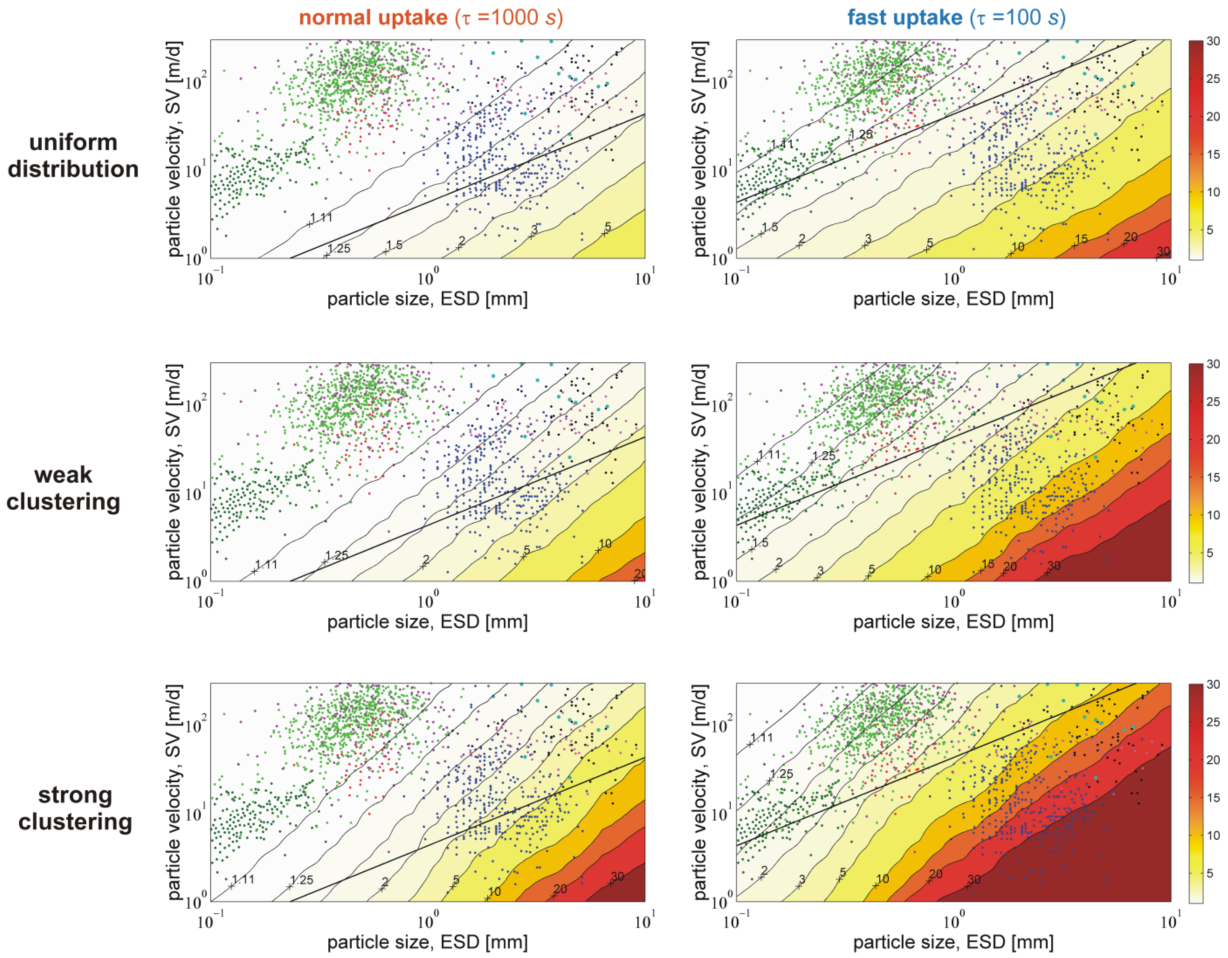

Figure A. Volume quenching factor for marine aggregates. Predicted impact of the bacterial uptake strength and degree of clustering on the volume of the trailing plume behind slow-sinking marine particles. The color represents the *volume quenching factor*, $E_V = V_{plm}^0 / V_{plm}$, which is defined as the ratio of the undisturbed plume volume, V_{plm}^0, at zero-uptake (Da=0) over the plume volume, V_{plm}, at any given conditions. The points represent experimental data for the sinking velocity (SV) and the equivalent sphere diameter (ESD) of individual aggregates. The straight black line corresponds to the timescale condition of Pe/Da=100 [*Kapellos*-2022]. Computations were performed for small organic solutes, like amino acids and oligo- saccharides, with a diffusivity of $\widetilde{D}_{Av} = 10^{-5}\ cm^2/s$. The contours correspond to selected values of the quenching factor (%relative change): 1.11 (10%), 1.25 (20%), 1.5 (34%), 2 (50%), 3 (67%), 5 (80%), 10 (90%), 15 (93%), 20 (95%), and 30 (97%). Source of experimental data: [*Bach*-2012] dark green, [*Nguyen*-2020] green, [*Iversen*-2020] purple, [*Nowald*-2009] red, [*Diercks*-1997] blue, [*Alldredge*-1988] magenta, [*Zetsche*-2020] dark cyan, and [*Cornec*-2015] black.

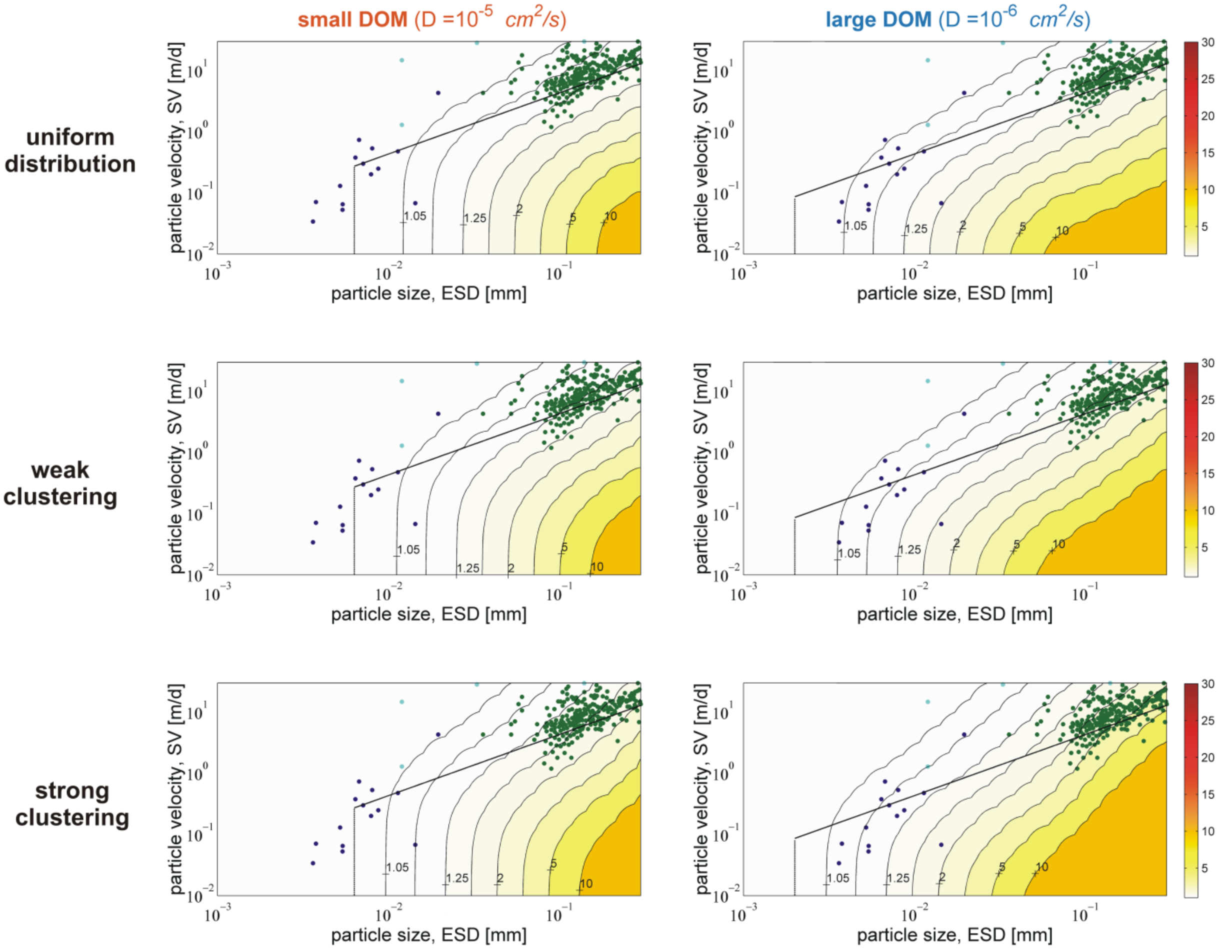

Figure B. Volume quenching factor for phytoplankton and small aggregates. Predicted impact of bacterial clustering on the volume of the eutrophic plume around small marine particles. The color represents the volume quenching factor. The points represent experimental data for the sinking velocity (SV) and the equivalent sphere diameter (ESD) of individual phytoplankton cells (dark blue [*Bach*-2012], cyan [*Karp*-1996]) and small aggregates (dark green [*Bach*-2012]). The straight black lines correspond to the timescale conditions of Pe/Da=100 and Da=10^{-4} that delineate the balance between uptake and advection or diffusion, respectively [*Kapellos*-2022]. Computations were performed for fast uptake, $\tilde{\tau}_U = 100s$, with a diffusivity of $\widetilde{D}_{Av} = 10^{-5}\, cm^2/s$ for small DOM (amino acids, oligo-saccharides) and a diffusivity of $\widetilde{D}_{Av} = 10^{-6}\, cm^2/s$ for large DOM (proteins, polysaccharides). The contours correspond to selected values of the quenching factor (%relative change): 1.05 (5%), 1.11 (10%), 1.25 (20%), 1.5 (34%), 2 (50%), 3 (67%), 5 (80%), and 10 (90%). For phytoplankton, experimental [*Smriga*-2016] and computational [*Bowen*-1993] studies have reported significantly stronger clustering, with β_m up to 100, that would result in more pronounced reshaping. However, we opted to use conservative estimates for the RDF parameters so as to to capture a wide range of particle types and avoid overrating the phenomenon.

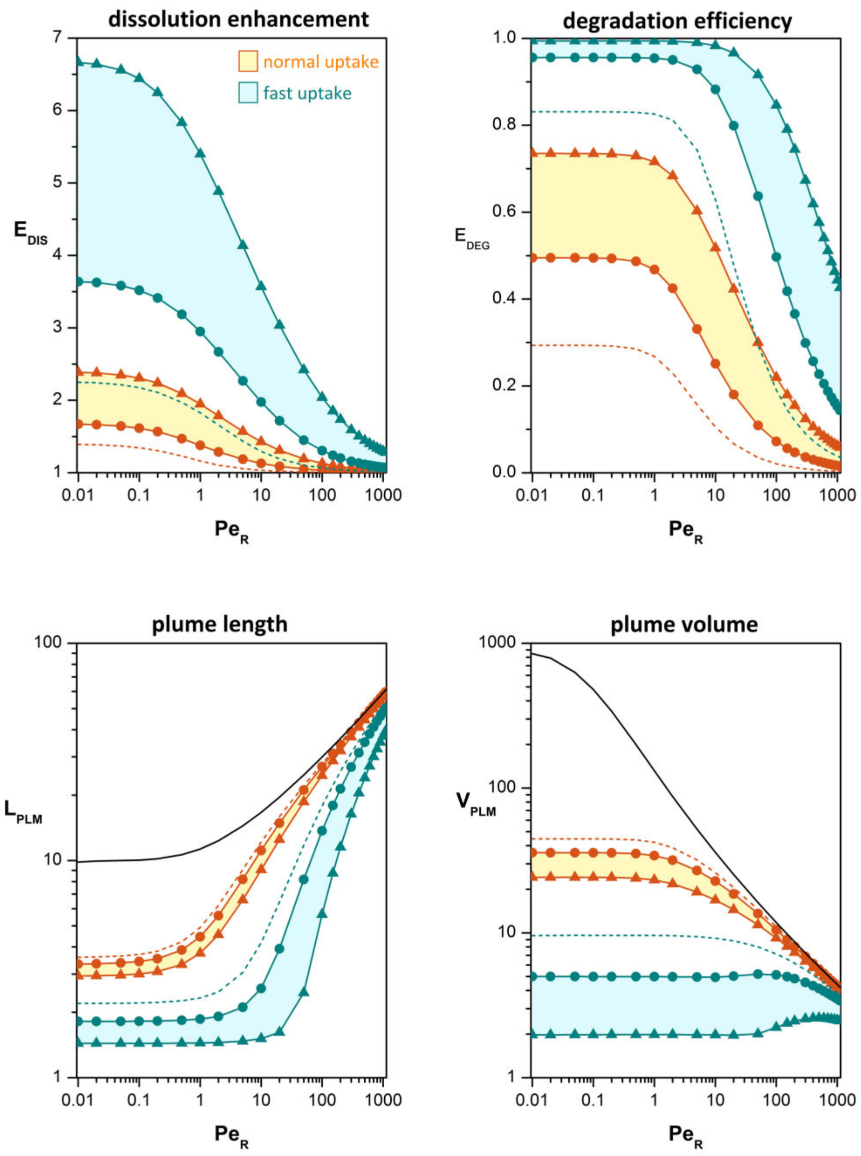

Figure C. Impact of bacterial microzones on plume metrics. The symbols represent the degree of clustering with circles for weak clustering, and triangles for strong clustering. The color represents the uptake strength with orange for normal uptake (Da=0.16), and cyan for fast uptake (Da=1.6). The black lines are undisturbed plume metrics (Da=0), and the short-dashed lines show the base effects for uniformly distributed bacteria. For normal uptake, the degree of clustering has an appreciable effect on the degradation rate, but a rather mild effect on plume metrics. This seemingly contradictory outcome is caused by the partial counterbalancing of consumption (E_{deg}) by enhanced dissolution (E_{dis}), under partition equilibrium at the particle surface. For fast uptake, the degree of clustering affects significantly all metrics, even if Pe/Da>100.

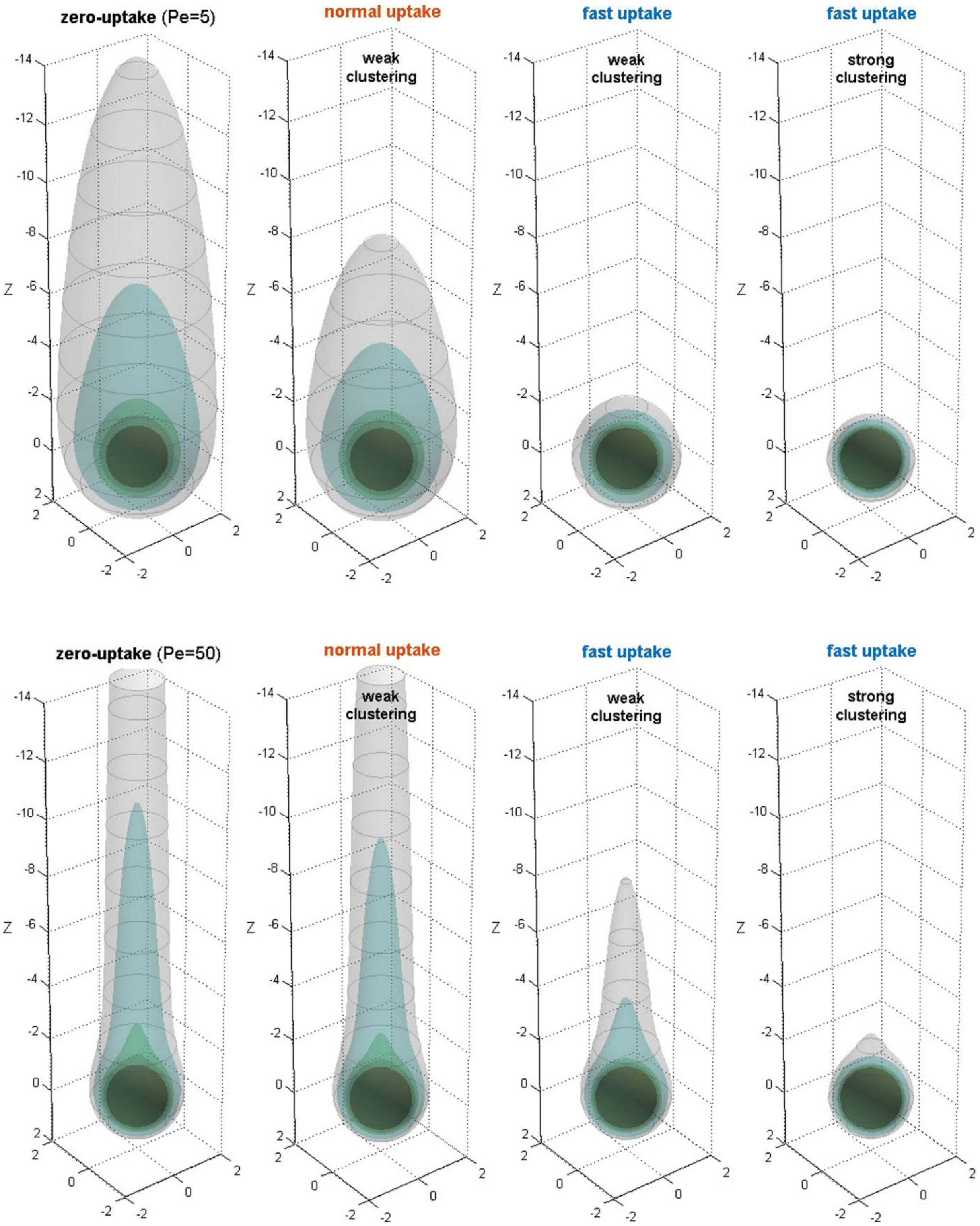

Figure D. Three-dimensional plume quenching. Slow-sinking particles at 1.1m/d (Pe=5, top) and 11m/d (Pe=50, bottom), under conditions of zero-uptake ($Da = 0$), normal uptake with weak clustering ($Da = 0.16$), and fast uptake ($Da = 1.6$) with weak or strong clustering (RDF parameters in Table 1). Nested isoconcentration surfaces are shown at selected values of nutrient concentration ($C_{A\upsilon}$ = 0.1, 0.2, 0.5, 0.7). The Péclet number corresponds to an alginate particle of radius $\tilde{R}_P = 0.4mm$ and oligo-alginate diffusivity of $\widetilde{D}_{A\upsilon} = 10^{-5}\ cm^2/s$ [*Alcolombri*-2021].

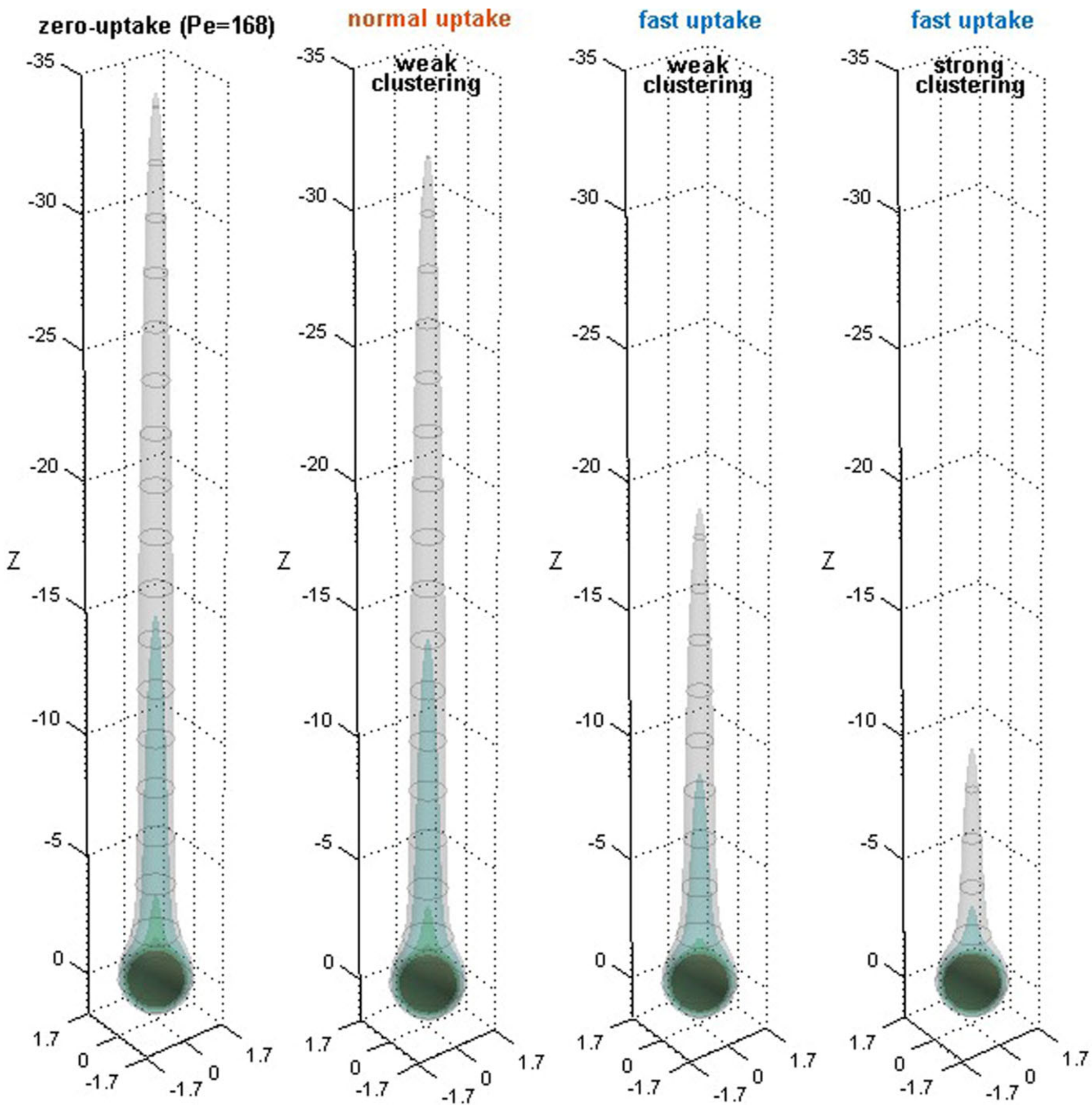

Figure E. Three-dimensional plume quenching (cont'd). Slow-sinking particle at 36.3m/d (Pe=168). The conditions of uptake and clustering are the same with Figure D.
