# Supplementary material for: Bacterial clustering amplifies the reshaping of eutrophic plumes around marine particles: A hybrid data-driven model
Source: PLoS Comput Biol. 2024 Dec 11;20(12):e1012660. doi: 10.1371/journal.pcbi.1012660 (PMC11666058; doi:10.1371/journal.pcbi.1012660)
Supplement: S1 Table — (PDF) [file pcbi.1012660.s004.pdf]

S1 Table. Characteristics of particle classes. Data for the number of individual particles, the particle size (ESD in mm) and sinking velocity (SV in m/d), and the relative change in plume length (% ΔL) under various uptake/clustering conditions for the three representative particle classes: large particles with low-to-moderate velocity (ESD>0.8mm, SV<100m/d), medium-sized particles with moderate-to-high velocity (0.3mm<ESD<0.8mm, 20m/d<SV<300m/d), and small particles with low velocity (ESD<0.3mm, SV<20m/d). Here, s.d. is the standard deviation, P10 and P90 are the 10th and 90th percentiles, respectively (i.e., 80% of the data fall in the P10-P90 interval).

	**SMALL**	**MEDIUM**	**LARGE**
ESD range	ESD < 0.3mm	0.3mm < ESD < 0.8mm	ESD > 0.8mm
SV range	SV< 20 m/d	20 m/d < SV <300 m/d	SV < 100 m/d
number of points	204 points	657 points	459 points
ESD<br>average (± s.d.)<br>[min, max]	 0.15 mm (± 0.06 mm)<br>[0.036 mm, 0.30 mm]	 0.50 mm (± 0.13 mm)<br>[0.31 mm, 0.80 mm]	 2.74 mm (± 1.88 mm)<br>[0.81 mm, 14.9 mm]
SV<br>average (± s.d.)<br>[min, max]	 9.6 m/d (± 4.7 m/d)<br>[1.2 m/d, 19.9 m/d]	 121.3 m/d (± 64.5 m/d)<br>[20.6 m/d, 295.7m/d]	 31.8 m/d (± 26.3 m/d)<br>[1.28 m/d, 98.1 m/d]
%ΔL<br>avg. [P10–P90]<br>normal/uniform<br>normal/weak<br>normal/strong<br>fast/uniform<br>fast/weak<br>fast/strong	 <br>1.71% [0.84–2.52] %<br>2.18% [1.11–3.26]%<br>3.33% [1.64–5.20]%<br>14.5% [7.80–20.2]%<br>17.8% [10.1–25.2]%<br>24.9% [14.3–36.7]%	 <br>1.40% [0.69–2.50]%<br>2.10% [1.10–3.74]%<br>4.26% [2.25–7.14]%<br>11.8% [6.40–19.8]%<br>17.3% [9.75–28.9]%<br>33.3% [20.4–51.6]%	 <br>26% [7.5–48.8]%<br>37% [11.1–70.5]%<br>60% [22.8–91.4]%<br>71% [43.4–92.3]%<br>85% [64.3–97.1]%<br>95% [90.4–98.3]%
