# Supplementary material for: Bacterial clustering amplifies the reshaping of eutrophic plumes around marine particles: A hybrid data-driven model
Source: PLoS Comput Biol. 2024 Dec 11;20(12):e1012660. doi: 10.1371/journal.pcbi.1012660 (PMC11666058; doi:10.1371/journal.pcbi.1012660)
Supplement: Attachment — Submitted filename: 2024-08-27 response_to_reviewer.pdf [file pcbi.1012660.s005.pdf]

**Authors' response to Reviewer #1:**

**Reviewer #1:** The manuscript presents an analysis of microbial quenching of nutrient plumes that form from particulate matter in marine environments. It builds on the model framework and analysis described in a previously published article (Microorganisms 2022) by the authors. In the original paper the bacterial distribution was considered uniform. In this new work the microbial population is modeled using a radial distribution function (RDF), which lumps together flow and chemical fields, chemotaxis, hydrodynamic interactions and particle-surface interactions. The RDF is based on data (experimentally and computationally derived) that were mined from existing literature to fit model parameters. An advection-dispersion-reaction model is used to describe nutrient concentration in the plume. The effect of two factors on plume quenching, clustering due to chemotaxis and fast nutrient uptake, were quantified in phase diagrams.

We thank the Reviewer for taking the time and effort to read very carefully our manuscript and, also, our previous paper upon which this work is built. We feel that their constructive comments helped us improve substantially our manuscript (MS).

Qualitatively, it makes sense that greater clustering and slower uptake would impact the plume to the greatest extent. However, the quantitative analysis is critical for truly gauging its relevance to microbial impact on the nutrient plumes from settling particles. The analysis suggests that only under conditions of strong clustering and fast nutrient uptake do the microorganisms impact the extent of the plume. The authors make the case that copiotrophs (e.g. Vibrio) operate under such conditions. The authors related different parametric regimes (particle size, sinking velocity, uptake rate and clustering) to specific marine microorganisms and the extent of plume quenching. I found this aspect to be a significant strength of the manuscript.

We thank the Reviewer for highlighting our parametric analysis and its relevance to specific marine microorganisms. We have further improved the corresponding sections in accordance with the provided technical and editorial comments. In the revised MS, we quantify more precisely the extent of plume quenching for different parametric regimes.

**Technical comments**

**1.** In Fig 1B the RDF model for strong clustering overestimates the bacterial concentration for all the data and the weak clustering model overestimates the Barbara data and a good portion of the Desai (r>1.5) and Smriga (r>2.2) data. To what extent does this then skew the stated importance of microbial reshaping of the plume?

We thank the Reviewer for highlighting this important point. In Fig 1B, each RDF dataset is normalized by a different average bacterial concentration and the RDFs with lower normalized values (Desai/Barbara) are associated with higher average concentrations (~$10^9$ cells/mL) due to the *observation window effect* that is discussed below. Therefore, the observed overestimation is an artifact caused by a bias towards very low normalized values of the ambient concentrations. We have considered this effect in the definition of the strong/weak clustering models and, thus, the stated importance of plume reshaping remains valid.

In particular, our RDF model is the superposition of a constant (ambient) value and an exponentially decreasing (excess) function. To accurately determine all RDF parameters, the datasets must have sufficiently large observation window. The size of the observation window, $\tilde{R}_\infty$, is the radial distance up to which data are provided for the bacterial concentration. If the observation window is smaller-than-required, then only the exponential part is viewed and the average bacterial concentration ($\tilde{B}_{avg}$) is overestimated (see p. S1-7 of the revised Supplement). This, in turn, leads to underestimation of the <u>normalized</u> peak and ambient concentrations ($\beta_m = \tilde{b}_m/\tilde{B}_{avg}$ and $\beta_0 = \tilde{b}_0/\tilde{B}_{avg}$). For the analyzed datasets, we have:

| data source | observation window, $\tilde{R}_\infty : \tilde{R}_P$ | average conc. $\tilde{B}_{avg}$ [cells/mL] | local ambient conc. $\beta_0 = \tilde{b}_0/\tilde{B}_{avg}$ | uptake timescale, $\tilde{\tau}_U = (\tilde{a}_S \tilde{B}_{avg})^{-1}$ |
|---|---|---|---|---|
| oil droplet (Desai, 2018) | 80μm:20μm (**4.0**) | 3.1x$10^9$ | 0.06 | 0.3 |
| amino acid bead (Barbara, 2003) | 100μm:32μm (**3.1**) | 1.9x$10^9$ | 0.07 | 0.5 |
| fecal pellet (Smriga, 2016) | 280μm:80μm (**3.5**) | 3.7x$10^6$ | 0.35 | 270.0 |
| phytoplankton (Bowen, 1993) | 620μm:20μm (**31**) | 1.0x$10^6$ | 0.96 | 1000.0 |

The Desai/Barbara datasets are biased towards very high average bacterial concentrations due to the small observation windows used in the **sources** of those data (thus, we cannot improve them). This results in severe underestimation of the ambient concentrations and, in turn, to the visual artifact noticed by the Reviewer in Figure 1B. What is more, the corresponding uptake timescales are extremely short (< $1\ s$, with $\tilde{\alpha}_S = 1\ pL/cell \cdot s$), which also implies that certain parameters ($\beta_0$, $\tilde{\tau}_U$) from these two datasets are unreliable and must be ignored.

<u>To avoid confusion and possible misinterpretation:</u>

- We updated Fig 1B to present only the excess part of the normalized bacterial concentration. It is thus shown that our analysis drills through the parameter space and the two clustering scenarios are indicative (strong/weak), rather than fully representative (strongest/weakest).
- We clarified that each RDF dataset is normalized with a different average bacterial concentration and listed the corresponding values in Table 1.
- We clarified that the strong/weak clustering models are parameterized within the range of extracted values for the peak bacterial concentration ($\beta_m$) and the chemotactic accumulation length ($d_s$), while the biased/unreliable ambient values ($\beta_0$) are ignored.

<u>Given this opportunity, we improved the RDF extraction procedure in SI through:</u>

- implementation of **constrained** regression analysis that incorporates information about the average bacterial concentration and, also, allows for fractional exponents.
- demonstration of the RDF extraction for a synthetic dataset generated with an individual-based simulator of bacterial chemotaxis.

**2.** Table 1 – Parameters for the algal cell (Bowen) seem to be outliers compared to the other 3 data sets listed. Are they consistent with known mechanistic differences for the plume formation? For example, does it make a difference that the algae are actively swimming rather than sinking.

We thank the Reviewer for raising this important point. As explained in detail below, the parameters for the algal cell (Bowen) stand out due to differences in the underlying mechanisms and the experimental setups. The striking difference in the chemotactic accumulation length ($d_s$), which is 2-5 times larger for the Bowen than other data, is rooted in chemotactic features (swimming speed, average run time, chemoreceptor saturation) that favor the formation of wider microzones by bacteria with run-and-tumble motility. The high values of the normalized peak and ambient concentrations ($\beta_0$ and $\beta_m$) are also attributed to the large observation window used by Bowen. We added a concise discussion on this point in the new section "Chemotaxis and RDF parameters" of the revised MS.

To shed more light on this discrepancy, we provide here a detailed comparison between the individual-based (IBM) simulations of Desai and Bowen. There are significant differences with regard to the flow and dissolution regimes, the chemotactic strength, and the setups.

| | **Desai, 2018** | **Bowen, 1993** |
|---|---|---|
| **Flow regime** | uniform flow past sphere | shear flow past sphere |
| **Dissolution** | transport-limited | reaction-limited |
| **Chemotaxis** | IBM (run-and-tumble) | IBM (run-and-tumble) |
| **Boundary conditions** | adsorption/depletion (open) | reflective (closed system) |
| **Observation window** | 80μm:20μm (**4.0**) | 620μm:20μm (**31.0**) |

The flow regime is a key difference. *Bowen et al.* considered shear flow past a sphere as an appropriate representation of the microscale flow field at the single-cell scale under macroscopic turbulence. Shear flow results in a motionless central core of size ~sqrt($D/E$) around the algal cell, in which nutrient transport is dominated by diffusion. Here, $D$ is the nutrient diffusion coefficient and $E$ is the fluid shear rate. Microzone formation is favored by such shear flow patterns (see Fig 1 in *Bowen et al.*) because the bacterial motion in the vicinity of the algal cell is undisturbed by flow. By contrast, *Desai et al.* considered uniform flow past a sphere as a proper representation of the flow regime around rising oil droplets (or sinking marine snow).

There are three other important differences between the computational setups of *Bowen* and *Desai*. First, *Bowen* considered reaction-limited dissolution (i.e., constant nutrient flux at the particle surface) whereas *Desai* considered transport-limited dissolution (i.e., constant nutrient concentration at the particle surface). Second, the number of bacteria in the computational domain is maintained constant in the simulations of *Bowen* (reflective boundary conditions), while it decreases over time in the simulations of *Desai* (bacteria are allowed to exit the domain and attach on the particle). Finally, *Bowen* used a much larger observation window than *Desai*, which affects the estimation of ambient bacterial concentrations ($\tilde{b}_0$ and $\tilde{B}_{avg}$).

**Nonetheless**, for both of the analyzed datasets, flow effects are weak and hence the impacts of dissolution and transport are also minimal. The IBM simulations are also similar, based on extensions of the classic chemotaxis model by *Brown & Berg* [PNAS, 71:1388-1392, 1974] and, in both studies, bacteria perform run-and-tumble motility without chemokinetic modulation of their speed. However, there are significant differences in the chemotaxis parameters. The wider microzones in the simulations of *Bowen* are attributed to two factors. First, *Bowen* used a significantly lower $C_0/K_D$ ratio than *Desai*, which inducts a weaker chemo-attractant gradient and less (chemotaxis-induced) directed motility towards the particle. Second, *Bowen* used a higher swimming speed and longer run time, which result in longer run lengths with reduced ability to maneuver and focus in a tight region around the chemoattractant source. Between tumbles, on average, bacteria run about 40μm in *Bowen*'s simulations (i.e., one particle diameter) and only 6μm in *Desai*'s simulations.

| | **Desai, 2018** | **Bowen, 1993** |
|---|---|---|
| **swimming speed, $V_S$** | 10 μm/s | 40 μm/s |
| **average run time, $\tau_0$** | 0.6 s | 1.0 s |
| **$C_0/K_D$ ratio*** | 1.00 | 0.015 |

* The parameter $C_0/K_D$ is the ratio of the average concentration on the particle surface over the chemoreceptor half-saturation of the specific bacteria.

As a final remark, the fact that data from microscale experiments (Barbara/Smriga) are closer to the simulations of *Desai*, may either be coincidental or perhaps imply that the *Desai* simulations are closer to realistic conditions. However, we need more high-quality datasets from single-cell simulations and experiments to reach firm conclusions on this issue.

**3.** I would expect the functional form and values of the fitting parameters for the RDF model to provide insight to the underlying processes that are dominating the bacterial distributions in the microzone. Please elaborate on any insight gained from determining the exponential functional dependence and magnitude of the parameters in the RDF model. For example, does a larger value of *n* imply a stronger chemotactic response? If so, is that consistent with the strength of the chemotactic response for the algal cell in the Bowen work compared to the other sources?

We thank the Reviewer for raising this excellent point. The available datasets, although rather limited and from disparate sources/systems, support the following observations.

First of, the good fit to the data by a simple exponential with maximum at the particle surface implies that there is no inhibition by the chemoattractant or any competing gradients from other chemoattractants or repellants that could result in more complex patterns, such as band formation. In future work, if datasets with significant deviations from exponential RDFs are available, the data could be approximated with spline RDFs that are highly versatile.

Second, the RDF parameters for the Desai, Barbara and Smriga datasets show that the chemotactic accumulation length ($d_s$) decreases as the normalized peak concentration ($\beta_m$) increases. This trend may be attributed to two different chemotaxis mechanisms. For chemotactic bacteria with run-and-tumble motility, the accumulation length decreases and the peak concentration increases (i.e., thinner and denser microzones) as the bacterial swimming speed decreases (new Figure 2 of the Revised MS). In this type of motility, high swimming speeds result in long run lengths and, thus, in reduced ability to maneuver and focus close to nutrient sources. By contrast, for chemokinetic bacteria with run-reverse motility and modulation of the swimming speed, the opposite trend has been observed [*Son et al.*, PNAS, 113:8624-8629, 2016]. That is, the microzone becomes thinner and denser as the average swimming speed increases because chemokinetic bacteria fine tune their speed and, thus, increase their ability to maneuver as they approach a chemoattractant source.

Moving forward, a systematic correlation between the RDF parameters and underlying mechanisms presents an exciting topic of future research. Here, in a preliminary step, we analyzed additional datasets from *Bowen*'s computational experiments. Extracted parameters (from datasets in Figure 2A of *Bowen*) support the aforementioned trend for the swimming speed effect on the accumulation of bacteria with run-and-tumble motility around nutrient sources (i.e., as the bacterial swimming speed increases, $d_s$ increases and $\beta_m$ decreases). One step further,

extracted parameters (from datasets in Figure 2C of *Bowen*) show that the exponent (*n*) is positively correlated to the normalized exudation rate, and higher exponent (*n*) is associated with wider and denser microzones (i.e., higher $\beta_m$ and higher $d_s$). The normalized exudation rate, $F^*$ [μm·s] in *Bowen*'s notation, captures the combined effects of the algal nutrient release and the bacterial chemotactic response. The nutrient release rate affects the thickness of the concentration boundary layer around the algal cell, while the chemotaxis mode and parameters weigh in the ability of the bacteria to maneuver and focus within the nutrient-rich layer. For given flow conditions, thicker boundary layers support thicker microzones.

We added a detailed discussion and additional results in the new section "Chemotaxis and RDF parameters" of the revised MS.

**4.** In Fig 3, provide more guidance for the reader to interpret the information presented in this phase diagram. It appears that only large particles with low-to-moderate sinking velocity and fast uptake kinetics are susceptible to plume quenching by marine microorganisms.

We thank the Reviewer for raising this point. We rephrased and clarified the discussion of Fig 3 (Fig 5 in the revised MS; also in accordance with the editorial comments #8 – #11).

In the revised MS, we define three representative particle classes: large particles with low-to-moderate sinking velocity (ESD > 0.8mm, SV<100m/d), medium-sized particles with moderate-to-high sinking velocity (0.3mm < ESD < 0.8mm, 20m/d<SV<300m/d), and small particles with low sinking velocity (ESD < 0.3mm, SV<20m/d). To provide accurate metrics for the predicted degree of quenching for each particle class, we carried out calculations for the available datasets {particle size, sinking velocity} of individual aggregates and we presented the detailed statistical output in S1 Table and new Figure 6 (box chart) of the revised MS.

Large particles are always susceptible to plume reshaping. For uniformly distributed bacteria with normal uptake kinetics, the plumes around large particles may be quenched by 10% to 50% in length/volume. The predicted degree of quenching is strongly amplified by clustering and fast uptake. On the other hand, medium and small particles become susceptible to reshaping only with elevated uptake kinetics. For example, for medium-sized particles, the average relative change in plume metrics ranges from 1.4% (no clustering) to 4.2% (strong clustering) with normal uptake kinetics, while the relevant range is from 12% (no clustering) to 36% (strong clustering) with fast uptake kinetics.

**Editorial comments**

**1.** L24 As written, this statement does not convey that the high potential for reshaping is limited to conditions of higher than normal uptake, which are typically associated with copiotrophs under a feasting scenario. Please rephrase to indicate that the high potential for reshaping applies to only certain conditions.

We rephrased this statement to reflect the conclusions more precisely.

**2.** L97-99 Cite the sources of the data sets that are mentioned.

We added the citations of the analyzed datasets.

**3.** L157 & 161 Include that C is also a function of x.

We made this clarification.

**4.** L180 Is the plume lifetime range from several seconds to tens of minutes rather than tenths of minutes? As stated, plume lifetimes from several seconds (e.g. 3 s) to tenths of minutes (e.g. 0.1 x 60s/min = 6 s) are nearly comparable values.

We thank the Reviewer for catching this typo. We meant "tens" and made the correction.

**5.** L185 Please clarify how chemotaxis is included in the terms for Eq (1). Does chemotaxis (clustering) increase BM and decrease dS? Then the hotspot metric is derived from these parameters using B(x). Is that correct? Or, do you start with an expected value for the hotspot metric and infer the parameter values for Eq (1)?

We thank the Reviewer for raising this point. We added the clarification.

Indeed, chemotaxis is included through the RDF parameters, and stronger chemotactic response is associated with higher microzone peak concentration ($\beta_m$) and shorter chemotactic accumulation length ($d_s$). The microzone radius ($R_M$) and hotspot index ($h_e$) are metadata, which are calculated once the RDF parameters have been determined by nonlinear regression.

For the parametric analysis, we fine tuned the parameters ($\beta_m$, $d_s$) so as to attain the same microzone radius and different hotspot index, while capturing the trend in the nutrient exposure as a function of the Péclet (Fig 3 in the Revised MS).

**6.** L188-191 The given ranges for the average bacterial abundance and affinity yield uptake timescales that range from 10^2 s to 10^10 s. Perhaps, the authors meant for the text to read tens of femtoliters rather than tenths of femtoliters. Even so, these ranges do not yield times of less than 100 s, so the statement that the uptake timescale may range from tenths to hundreds of seconds is incorrect. I noticed that this error was carried over from a previous publication (Microorganisms 2022).

We thank the Reviewer for catching this typo. We meant "tens" and made the correction.

The stated range for the uptake timescale (tens to hundreds of seconds) has been calculated for bacterial abundances (10^6 to 10^7 cells/mL) and nutrient affinities (1 to 10 pL/cell*s), on the typical-to-high end of values for particulate blooms. So, given that $\tilde{\tau}_U = 1/\tilde{k}_U$, with $\tilde{k}_U = \tilde{a}_S \tilde{B}_{avg}$, we obtain the following range:

slow uptake: $\tilde{k}_{U,slow} = \left(\frac{1\,pL}{cell\cdot s}\right)\left(\frac{10^6 cells}{mL}\right) = 10^{-3} s^{-1}$ and $\tilde{\tau}_{U,slow} = 10^3 s$

fast uptake: $\tilde{k}_{U,fast} = \left(\frac{10\,pL}{cell\cdot s}\right)\left(\frac{10^7 cells}{mL}\right) = 10^{-1} s^{-1}$ and $\tilde{\tau}_{U,fast} = 10\ s$

The ranges of values for the bacterial abundance and nutrient affinity are clearly stated in our previous publication (last paragraph on p.8 of [Microorganisms, 2022]), so there is no error there (apart from the typo "tenths"). In this manuscript, indeed, we inadvertently neglected to specify these ranges. We apologize for the confusion. We made the clarification in the revised MS.

Furthermore, timescales less than 100s characterize systems at the high end of bacterial abundances (10^7 cells/mL or higher) and nutrient affinities (1 pL/cell*s or higher), which may be encountered only during particulate blooms. For example, *Smriga et al.* (2016) provide parameters for the estimation of the uptake timescale for typical "coastal water" and "diatom bloom" conditions. Following their work (Eq. 1, p. 1577), the uptake timescale is estimated as:

$$\tilde{\tau}_U = 1/\tilde{k}_U \quad \text{with} \quad \tilde{k}_U = 4\pi\tilde{R}_{NM}\tilde{D}\tilde{B}_0 + 4\pi\tilde{R}_M\tilde{D}\gamma\tilde{B}_0$$

where $\tilde{D}$ is diffusion coefficient of the nutrient, $\tilde{R}_{NM}$ is the radius of non-motile bacteria, $\tilde{R}_M$ is the radius of motile bacteria, $\tilde{B}_0$ is the bulk concentration of non-motile bacteria, and $\gamma\tilde{B}_0$ is the bulk concentration of motile bacteria.

typical coastal seawater conditions:

$$\tilde{D} = 10^{-5} cm^2/s,\ \tilde{R}_{NM} = 0.2\mu m,\ \tilde{R}_M = 0.5\mu m,\ \tilde{B}_0 = 5\cdot 10^5 cells/mL,\ \gamma = 0.1$$

$\Rightarrow$ $\tilde{k}_U = 0.00157\ s^{-1}$ and $\tilde{\tau}_U = 636\ s$

typical diatom bloom conditions:

$$\tilde{D} = 10^{-5} cm^2/s,\ \tilde{R}_{NM} = 0.2\mu m,\ \tilde{R}_M = 1\mu m,\ \tilde{B}_0 = 10^7 cells/mL,\ \gamma = 0.1$$

$\Rightarrow$ $\tilde{k}_U = 0.0377\ s^{-1}$ and $\tilde{\tau}_U = 27\ s$

**7.** L192 Cite the source for the recently shown result.

We cited the source.

**8.** L215 Clarify what is meant by essential in the context, "particle classes for which plume reshaping is essential".

We clarified this vague statement. The word "meaningful" is used in place of "essential" to denote that the predicted degree of quenching may be >10%.

**9.** L216 In categorizing the particle classes, if low-to-moderate sinking velocity is classified as SV<100m/d, did the authors intend for moderate sinking velocity to be SV>100m/d? Otherwise, a large portion of the medium particles lie outside the criterion.

We clarified the definition of the representative particle classes.

**10.** L233-236 For which uptake strength does this description apply? It does not seem consistent with the panels in Fig 3. For example, for uniform distribution and fast uptake the medium sized particles mostly lie outside the EL=2 (50%) contour. Please clarify the description of the bounds for which quenching is important.

We clarified the conditions for which plume quenching is important.

**11.** L243-245 Cite the source for this statement.

Thank you for paying attention to this important detail. We cited the relevant source.
